# A recessive mutation in muscadine grapes causes berry color-loss without influencing anthocyanin pathway

**DOI:** 10.1038/s42003-022-04001-8

**Published:** 2022-09-24

**Authors:** Ahmed Ismail, Pranavkumar Gajjar, Minkyu Park, Abdulla Mahboob, Violeta Tsolova, Jayasankar Subramanian, Ahmed G. Darwish, Islam El-Sharkawy

**Affiliations:** 1 Center for Viticulture and Small Fruit Research, College of Agriculture and Food Sciences, Florida A&M University, Tallahassee, FL 32308 USA; 2 Department of Horticulture, Faculty of Agriculture, Damanhour University, Damanhour, Egypt; 3 Department of Chemistry, College of Sciences, United Arab Emirates University, Al-Ain, P.O. Box 15551, United Arab Emirates; 4 Department of Plant Agriculture, University of Guelph, P.O. Box 7000, Vineland Station, ON L0R 2E0 Canada; 5 Department of Biochemistry, Faculty of Agriculture, Minia University, Minia, 61519 Egypt

**Keywords:** Plant genetics, Metabolomics

## Abstract

Anthocyanins, a major class of flavonoids, are important pigments of grape berries. Despite the recent discovery of the genetic cause underlying the loss of color, the metabolomic and molecular responses are unknown. Anthocyanin quantification among diverse berry color muscadines suggests that all genotypes could produce adequate anthocyanin quantities, irrespective of berry color. Transcriptome profiling of contrasting color muscadine genotypes proposes a potential deficiency that occurs within the anthocyanin transport and/or degradation mechanisms and might cause unpigmented berries. Genome-wide association studies highlighted a region on chromosome-4, comprising several genes encoding glutathione S-transferases involved in anthocyanin transport. Sequence comparison among genotypes reveals the presence of two GST4b alleles that differ by substituting the conserved amino acid residue Pro_171_-to-Leu. Molecular dynamics simulations demonstrate that GST4b2–Leu_171_ encodes an inactive protein due to modifications within the H-binding site. Population genotyping suggests the recessive inheritance of the unpigmented trait with a GST4b2/2 homozygous. A model defining colorless muscadines’ response to the mutation stimulus, avoiding the impact of trapped anthocyanins within the cytoplasm is established.

## Abstract (web summary)

Transcriptome profiling and mutational analysis suggest a potential deficiency in anthocyanin transport by glutathione S-transferases and/or degradation mechanisms that might cause unpigmented berries.

## Introduction

In plants, flavonoids are synthesized through a branched pathway yielding different classes of flavonoid compounds. The major classes of flavonoids are anthocyanins (red to purple pigments), flavonols (colorless to light-yellow pigments), as well as flavanols and proanthocyanidins (PAs) or condensed tannins (colorless pigments that become brown after oxidation). These compounds are widely distributed in different amounts, according to the plant species, organ, developmental stage, and growth conditions^1^. They perform a wide range of functions, including plant development, reproduction, defense, and protection against abiotic stresses^2–4^. Among the most abundant classes of grape flavonoids, anthocyanins (ACNs) accumulate mainly in the berry skin, whereas PAs are located in both skin and seed^5^.

In grapes, anthocyanins are the typical end-products of phenylpropanoid metabolism. Six types of anthocyanins have been recognized in *Vitis* and *Muscadinia* grapes^6,7^. In *Vitis*, anthocyanins are acylated 3-*O*-monoglucosids and predominantly present in the malvidin form; however, *Muscadinia* produces non-acylated 3,5-*O*-diglucosidic anthocyanins with a majority of delphinidin type^7,8^. Recently, we have characterized the flavonoid biosynthetic pathway in muscadine, among which three major associated factors have been proposed to be involved in anthocyanin accumulation^9^. The first factor is the regulatory mechanism that coordinates the expression of anthocyanin biosynthesis genes during fruit development or in response to environmental stimuli^10^. Anthocyanin biosynthesis is synchronized by a transcription complex composed of two transcription factors in the R2R3-MYB and the bHLH-MYC protein families, and a WD40 co-factor protein. The three proteins co-function by forming the MBW-complex to activate the expression of a downstream cascade of structural genes in the anthocyanin pathway. Despite the critical contribution of WD40 in the complex, the MYB/MYC proteins are the key components in providing specificity for the subsets of biosynthetic genes and in determining color levels^11^. The second factor is the biosynthetic pathway, involving several enzymes that catalyze a sequential reaction for anthocyanin synthesis within the cytoplasm compartment. In muscadine, the expression of most genes involved in the anthocyanin biosynthetic pathway is positively correlated with anthocyanin accumulation^9^. However, in *Vitis* grapes, only the action of the UFGT enzyme has been shown as the critical limiting step in anthocyanin biosynthesis^12^. The third factor is the final step in the pathway, the anthocyanin transport. Two kinds of molecular actors are putatively involved in the vacuolar sequestration of anthocyanins, glutathione S-transferases (GSTs) and ATP binding cassette (ABC) that mediate anthocyanin/GSH co-transport^13^. Once anthocyanins are synthesized within the cytosol, they are transported to the site of permanent storage in the vacuole, and this localization is necessary for their stabilization^14^.

GSTs encode multigene family proteins that have been described to be essential for anthocyanin accumulation^15^. Gene knockout and complementation studies have demonstrated that GSTs (i.e., maize*-Bz2*, petunia*-An9*, carnation-*Fl3*, litchi-*LcGST4*, and *Arabidopsis*-*TT19*) are indelibly involved in anthocyanin transport^16–20^. The significance of GSTs originates from their function as carriers and vacuolar sequestration of metabolites, including anthocyanins^21^. The covalent glutathione (GSH) tag mediates the recognition of molecules destined for vacuolar sequestration by a tonoplast-localized ATP-binding cassette pump^17^. This simplified description suggested that all anthocyanin-related components should work in harmony for successful fruit pigmentation by which any disruption can cause color loss. Characterizing plant mutants exhibiting phenotypic alterations in their pigmentation program should help better understand the mechanisms through which anthocyanins are regulated, biosynthesized, and accumulated.

While *Vitis* grapes exhibit a broad spectrum of berry color, muscadines produce only two main color types, black/purple and bronze^7,22^. This initial observation suggested that the genetic basis underlying the diversity in the color trait is different between the two species. In *Vitis* grapes, the berry color locus is associated with a single MybA gene cluster on chromosome-2^23,24^. The ultimate berry color is the result of additive effects from alleles of 3 MYB-type transcription factors, VvMybA1, VvMybA2, and VvMybA3, within the single MybA gene cluster through which VvMybA1 and VvMybA2 were demonstrated to be functionally involved in berry pigmentation^25,26^. However, the quantitative diversity of berry color is due to polymorphisms within the MybA genes that caused structural changes in the MybA promoters and proteins^23,27^. Consequently, the loss of color character is inherited as a recessive trait and has been linked to the presence of a single gypsy-type retrotransposon in a homozygous form of the VvMybA1 promoter^23,27,28^.

Muscadine berry color is a critical trait in defining the quality of processed products. From consumers’ perspective, colored skin grapes provide essential cultivar differentiation and health benefits due to their bioactive properties^12,29^. In the current manuscript, a GWAS study was used to identify the origin of the loss-of-pigmentation trait in muscadine. However, biochemical and transcriptome approaches were applied to identify the changes in vine strategies in response to the mutation stimulus. Anthocyanin quantification among several muscadine genotypes proposed that all muscadines could produce anthocyanins, regardless of visible color. Transcriptome analysis suggested the involvement of two potential mechanisms in coordinating the diversity of muscadine berry color, anthocyanin transport and/or degradation. GWAS and HRM data confirmed that the berry color locus in *M. rotundifolia* was associated with a locus on chromosome-4 that contains a gene encoding glutathione S-transferases involved in anthocyanin transportation^30–32^. The loss of pigmentation character in muscadine was associated with the presence of a single substitution in the conserved amino acid residue Pro_171_-to-Leu in a homozygous form^31^. Dynamics simulation analysis indicated that GST4b2–Leu_171_ encodes an inactive protein due to considerable changes in the GSH/flavonoid binding site. Further, metabolome quantification and enzymatic assays suggested an enhanced anthocyanin degradation mechanism in unpigmented muscadine genotypes in response to the mutant stimulus.

## Results and discussion

### Color characteristics of muscadine grapes

To determine color characteristics in muscadine grapes, ten muscadine genotypes with varying visible berry colors at ripening were selected (Supplementary Fig. 1). The calculated colorimetric indexes highlighted the relevant differences among genotypes (Supplementary Table 1). The L*, a*, b*, hue angle (*H*), and chroma (*C*) values were in agreement with the data reported previously for muscadine grapes^32^. The analysis divided the 10 genotypes into two main groups of unpigmented (green/bronze) and colored (red/purple/black) berries. The unpigmented muscadine berries exhibited high values of luminosity-L* (36.9−42.8) and chroma-*C* (14.6–20.1) but with low hue-*H* values (16.9–38.5). Contrarily, the colored berries displayed low L* (18.8–23.6), *C* (1.0−7.9), and high *H* values (340.0–357.9).

Generally, the analysis of TAC and IAC levels in berry skin at ripening was consistent with the visual observation, excluding the Rosa genotype (Supplementary Table 2). The TAC and IAC levels were markedly higher in colored than in unpigmented genotypes^33^. As reported previously, HPLC analysis identified the delphinidin-3,5-diglucoside (DEL), cyanidin-3,5-diglucoside (CYA), petunidin-3,5-diglucoside (PET), peonidin-3,5-diglucoside (PEN), malvidin-3,5-diglucoside (MAL) as the only anthocyanins detected in muscadine berries^7^. In colored genotypes, the DEL showed the highest accumulated anthocyanin (29–72%); however, the other anthocyanins considerably accumulated but in a genotype-dependent manner^34^. In unpigmented genotypes, the CYA represented the major anthocyanin (47–91%), followed by the DEL (4–38%). The other anthocyanins were almost undetectable (0–13%). Despite the red color of the Rosa genotype (Supplementary Fig. 1), the berries displayed an anthocyanin accumulation profile comparable to unpigmented genotypes in terms of the type and quantity of anthocyanins (Supplementary Table 2; Supplementary Data 1). Interestingly, these results demonstrated that both colored and unpigmented muscadines produce adequate quantities of anthocyanins to visualize skin pigments, suggesting the presence of other unknown factors that cause pigmentation-loss phenotype in muscadine.

### Evaluation of anthocyanins and PAs traits during development

**Fig. 1:**
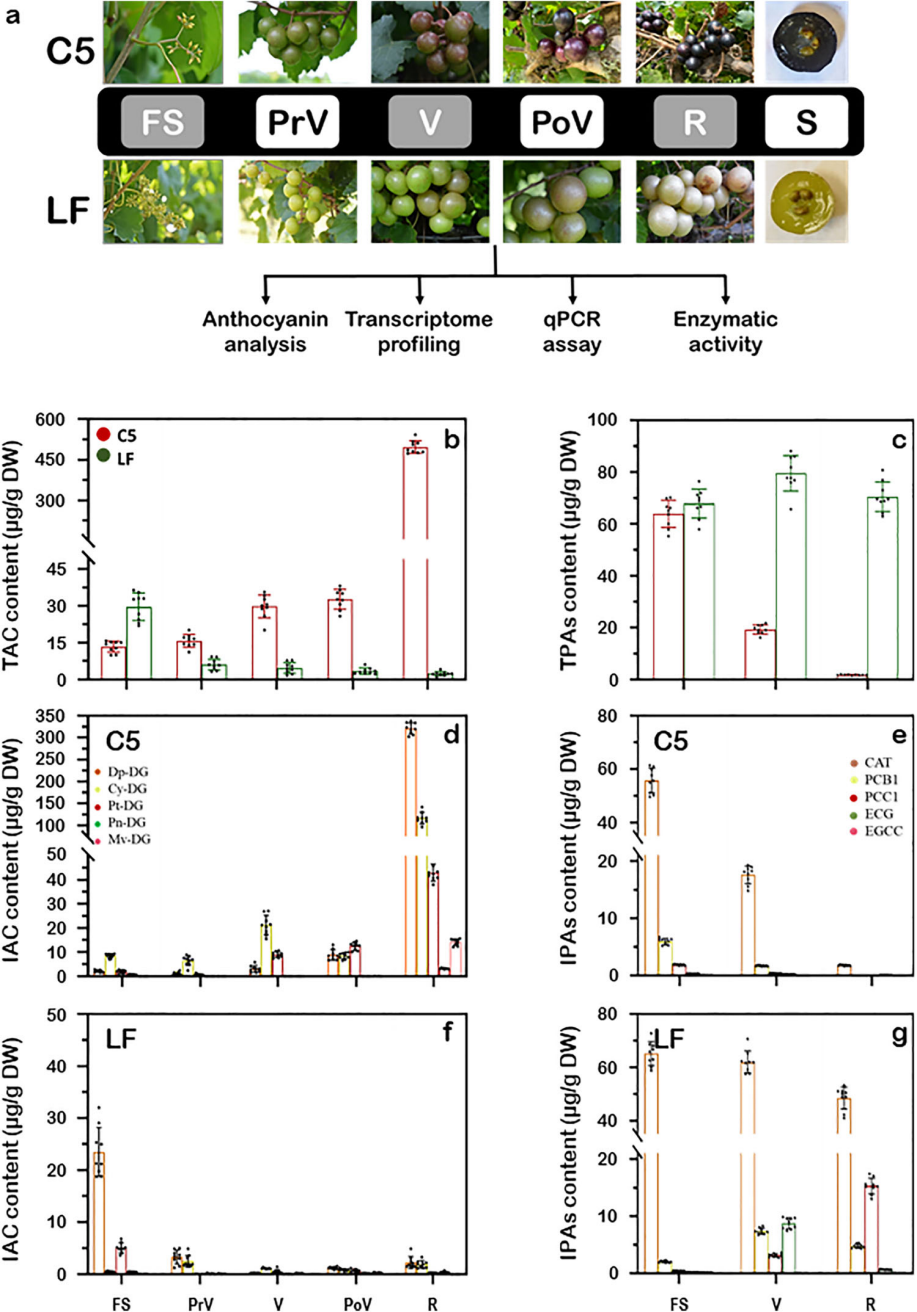
The layout of the study using the two-muscadine genotypes, C5 and LF, exhibits color discrepancy. **a** Developmental stages highlighted in gray represent the samples selected for RNA-seq, including fruit-set (FS), véraison (V), and ripening (R). However, the stages of pre-véraison (PrV), post-véraison (PoV), and seeds (S) were only included in the qPCR assay. The changes in total anthocyanin content-TAC (**b**), total proanthocyanidin content-TPAs (**c**), individual anthocyanin content-IAC (**d** and **f**), and individual proanthocyanidin content-IPAs (**e** and **g**) in the skin during C5 and LF berry development. Proanthocyanidins were assessed in FS, V, and R stages. Bars are the mean of three biological and technical replicates (*n* = 9; ±SD).

To determine the strategy of color development in muscadine berry, the total and individual anthocyanins and PAs were assessed in C5 and LF berries throughout development (Fig. 1a). In C5, the TAC and IAC levels were initially low at the FS stage and steadily increased with berry development, reaching maximal levels at ripening (Fig. 1b, d). The CYA was the major anthocyanin during the immature stages of FS (64.3%), PrV (61.8%), and V (61.9%). However, DEL (29.9%), CYA (28.7%), and PET (41.7%) were the main contributors to pigmentation at the PoV stage. At ripening, all IACs were considerably detected; however, the DEL was the predominant form (64.5%). Contrary to C5, the TAC and IACs in LF was relatively detected at the FS stage and gradually declined with development, reaching undetectable levels at ripening (Fig. 1d, f). Among different IACs, the DEL was the major accumulated form with a range of 36–79%.

Recently, an untargeted metabolome profile using UPLC–TOF–MS analysis identified the PAs—procyanidin C1 (PCC1), procyanidin B1 (PCB1), epigallocatechin 3-cinnamate (EGCC), epicatechin-3-gallate (ECG), and catechin (CAT)—in the berry skin of same muscadine samples used in current study^29^. HPLC analysis demonstrated that CAT was the major accumulated PAs form in muscadine berry skin with a range of 63–83% (Fig. 1e, g). In C5, the TPAs and IPAs levels exhibited a contradictory accumulation profile to anthocyanin (Fig. 1). They were abundantly detected at the FS stage and severely declined during development, reaching undetectable levels at ripening. Contrary to C5, TPAs and IPAs did not show a clear association profile with anthocyanin during LF berry development since they constantly accumulated at high levels (Fig. 1c, e, g). In the structural biosynthetic pathway, anthocyanin and PAs compete for the same leucoanthocyanidin precursor^35^. Our data suggested that the stimulation of a particular biosynthesis direction affects the other pathway and this should occur in a developmental stage- and genotype-dependent manners. At the immature stage, the PAs biosynthesis pathway is dominant, a pattern that declined along with development in colored genotypes. However, in unpigmented genotypes and due to the minor anthocyanin input, the PAs remained at constant high levels during development.

### Global changes in transcriptome profile during berry development

**Fig. 2:**
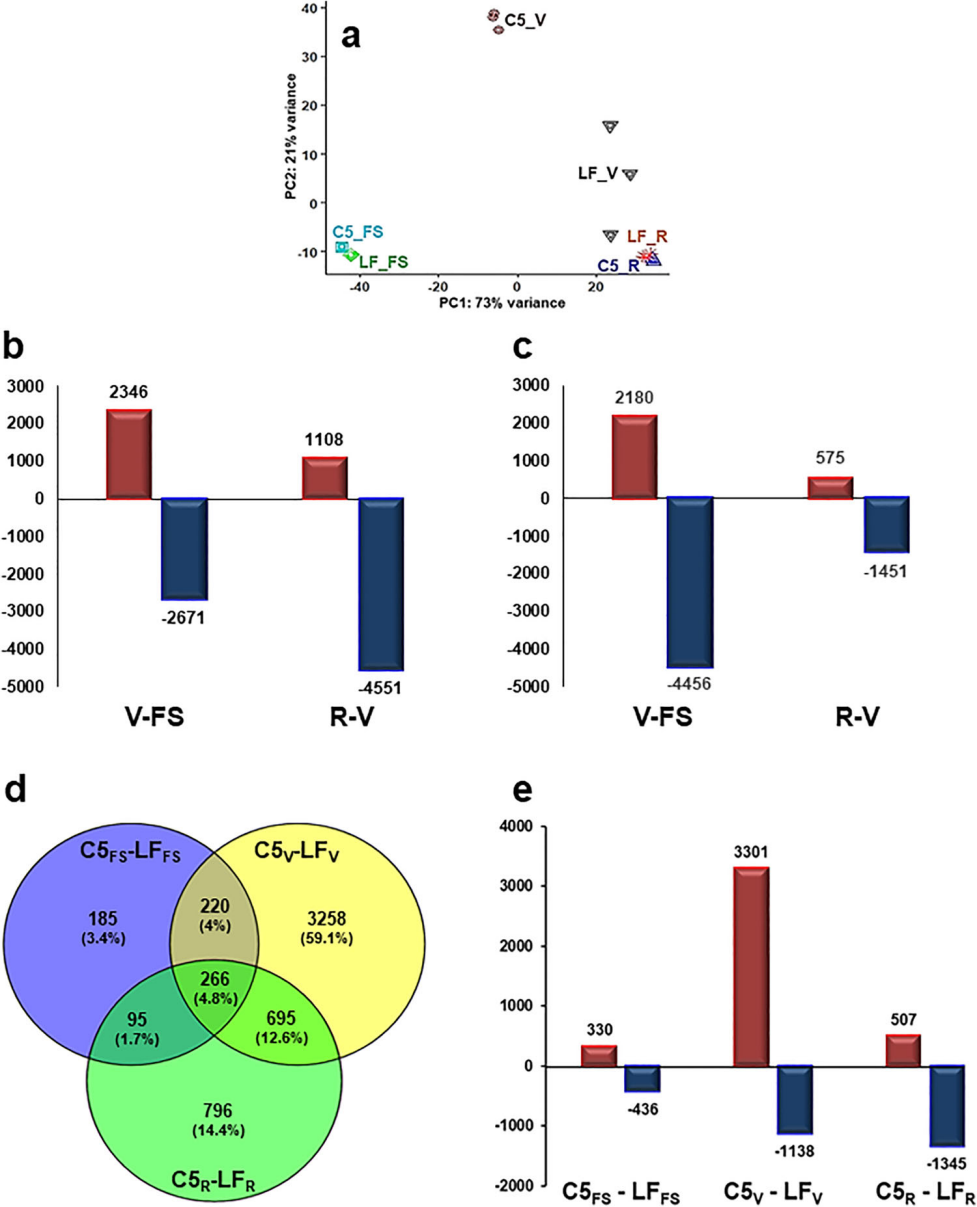
Transcriptome profiling during C5 and LF berry development. **a** The principal components analysis (PCA) of the samples’ replicates, where normalized counts were transformed to the variance stabilizing transformation (VST) using DESeq2. Each unique combination of a developmental stage was given a distinctive color. **b**–**e** By using DESeq2 and EdgeR pipeline, DEGs from each comparison were identified with fold2change >1.5 or <–1.5 (*P*-adjusted < 0.05). **b** and **c** Bar plot of differentially expressed genes (DEGs) in C5 and LF when each stage was compared to its former one. **d** and **e** Venn diagrams and bar plots of DEGs resulted from comparing equivalent stages in C5 and LF genotypes.

The differences between C5 and LF genotypes with contrasting berry color traits suggested potential molecular events that coordinate anthocyanin accumulation throughout berry development in a genotype-dependent manner. The RNA-seq libraries of FS, V, and R stages from C5 were analyzed versus their counterpart in LF. The total length of high-quality clean reads was 18.9–38.8 million among the 18 libraries, exhibiting 77.4–80.1% mapping rate against muscadine transcriptome (Supplementary Data 2). Principal component analysis (PCA) exhibited clear separation and consistency among berry developmental stages (Fig. 2a; Supplementary Fig. 2). The first two components could explain 94% of the variance in the transcriptome profiles, 73% (PC1) and 21% (PC2). The PC1 and PC2 were mainly associated with berry development and genotype diversity, respectively, where the V stage seems to be the main contradictory point between genotypes. Similarly, substantial alteration of the berry transcriptome profile was detected between colored and unpigmented genotypes as berry development proceeds, and changes in anthocyanin levels occurred^9,36^.

To identify differentially expressed genes (DEGs) during berry development, the transcriptome profiles of the developmental stages were compared (V–FS or R–V) within the same genotype (C5_stages_ and LF_stages_) or against applicable corresponding (C5_FS_–LF_FS_, C5_V_–LF_V_, and C5_R_–LF_R_) in both genotypes (C5_stage_–LF_stage_). Two statistical packages DESeq2 and EdgeR were utilized and only DEGs with high expression values (*P*_FDR_ < 0.05, log2fold change > 1.5 or <–1.5) in each pairwise comparison were considered^37,38^ (Supplementary Figs. 3 and 4; Supplementary Data 3–5). The four pairwise comparisons within C5_stages_ and LF_stages_ resulted in 8034 and 7412 non-redundant DEGs, respectively, where C5_V–FS_, C5_R–V_, LF_V–FS_, and LF_R–V_ resulted in 5017, 5659, 6636, and 2026 DEGs, respectively (Fig. 2b, c, e; Supplementary Fig. 5; Supplementary Data 3 and 4). However, the three C5_stage_–LF_stage_ transcriptome comparisons (C5_FS_–LF_FS_, C5_V_–LF_V_, and C5_R_–LF_R_) identified 766, 4439, and 1852 DEGs, respectively, and 5515 non-redundant DEGs in all comparisons (Fig. 2d, e; Supplementary Data 5). The data demonstrated that transcriptional reprogramming of a large number of genes is associated with berry development. Moreover, the number of up- and down-regulated DEGs significantly varied in both genotypes as berry development proceeds, particularly at the V stage that represents the main divergent point in transcriptome modulation between C5 and LF. For instance, the number of DEGs between FS and V slightly varied in C5 (C5_V–FS_; Fig. 2b), but substantially varied in LF where DEGs at V represent roughly 2-times of the number at FS stage (LF_V–FS_; Fig. 2c). Contrary, as berry development progresses, the number of DEGs at V comparing to R stage was ~4-fold higher in C5 (C5_R–V_; Fig. 2b), and only ~2.5-fold higher in LF (LF_R–V_; Fig. 2c). Consistency, ~3-fold of DEGs were up-regulated during the V stage in C5 comparing to LF (C5_V_–LF_V_; Fig. 2e). Contrary, LF_R_ stage accumulated more DEGs by ~2.7-fold relative to C5_R_. The data exhibited that 32.2% (3314 out of 10,285) of DEGs were common between the C5_stages_, LF_stages_, and C5_stage_–LF_stage_ (Supplementary Fig. 6). Additionally, the down-regulation pattern of gene expression dominated the up-regulation profile throughout berry development, especially at the V stage, as previously reported in different *Muscadinia* and *Vitis* genotypes^9,36,39^. These three analyses agreed with PCA data, emphasizing the V stage as a discriminating point where a dramatic transcriptome remodeling takes place within the same genotype or between genotypes. Consistently, the V stage is considered a crucial period where acidity decreases, but sugars and pigments (in colored genotypes) accumulated^40^. These DEGs encompass the most relevant genes for anthocyanin as they include not only DEGs between the two genotypes at three developmental stages but also DEGs between two adjacent developmental stages within C5 and/or LF, which account for color progression (in C5) that may not be captured by direct comparison between LF or C5.

### Identification of WGCNA modules associated with anthocyanin accumulation

**Fig. 3:**
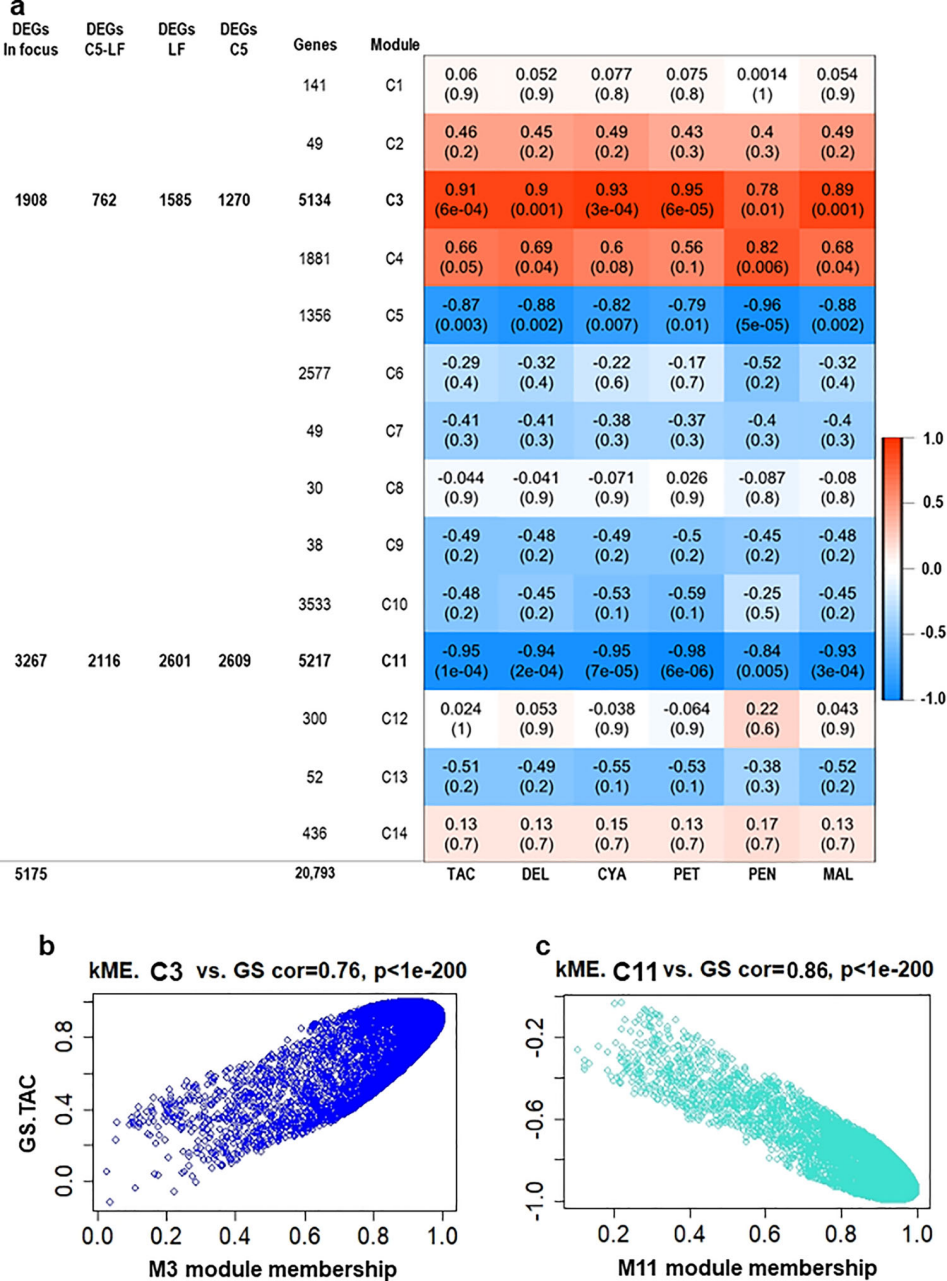
Module–trait associations and gene relationship to the trait in modules of interest. **a** Module–trait associations between RNA-seq data and anthocyanin-related traits, including TAC, delphinidin (DEL), cyanidin (CYA), petunidin (PET), peonidin (PEN), and malvidin (MAL) from C5 genotype throughout berry developmental stages. The correlation coefficient between a given module and traits is indicated by the color of the cell at the row–column intersection. Each row corresponds to a module (C1–C14). The left panel shows the assigned number of genes to each module, whether the number of total input genes or DEGs from C5_stages_, LF_stages_, and C5_stage_–LF_stage_ comparisons. DEGs from each comparison were identified with fold2change > 1.5 or <–1.5 (*P*-adjusted < 0.05). The modules of interest were shown in bold and were selected for further analysis (indicated as ‘In focus’). Blue and red are the color key that represents ‘*r*’ values. The gene significance in modules C3 (**b**) and C11 (**c**). The ‘GS.TAC’ is the gene significance with the TAC. The module eigengene connectivity (kME) was calculated for each gene within the C3 and C11 modules.

Anthocyanins are amongst the decisive players of fruit quality that undergo dramatic changes during berry development^41^. To elucidate molecular events associated with anthocyanin biosynthesis in the two contrasting genotypes, a system biology approach WGCNA was utilized^42^ (Supplementary Fig. 7). In fact, the berry transcriptome analysis of C5 and LF was of great significance to anthocyanin biosynthetic genes stimulated throughout berry development, mostly in the C5 genotype. Therefore, co-expressed gene sets were identified in colored C5, where the abundantly expressed genes across all developmental stages were examined^43^. The analysis of module–trait association among the RNA-seq data and the C5 anthocyanin property data, including TAC, DEL, CYA, PET, PEN, and MAL identified 14 modules (C1–C14; Fig. 3a; Supplementary Fig. 8). The two modules C3 and C11 were positively (*r* = 0.91, *P* = 6.0 × 10^−4^) and negatively (*r* = –0.95, *P* = 1.0 × 10^−4^) correlated in a significant manner with the TAC trait, holding 1908 and 3267 DEGs, respectively, which were assigned from C5_stages_, LF_stages_, and C5_stage_–LF_stage_ (Fig. 3; Supplementary Fig. 9; Supplementary Data 6). Based on the *V. vinifera* Ensembl GeneID, Gene Ontology (GO) terms, Kyoto Encyclopedia of Genes, and Genomes (KEGG) were searched in the DEGs of C3 and C11 modules^44^ (Supplementary Data 7). The GO enrichment analysis for eigengenes in positively correlated module C3 identified significantly enriched flavonoid-related biological processes (BPs) GO categories, such as phenol-containing compound metabolic process and phenol-containing compound biosynthetic process (Supplementary Fig. 10; Supplementary Data 7). The KEGG analysis of the C3-related DEGs provided further information about the enriched anthocyanin-related pathways, such as phenylalanine metabolism, flavonoid biosynthesis, biosynthesis of secondary metabolites, and phenylpropanoid biosynthesis. The last two KEEG pathways were also enriched in the C11-containing DEGs, as well as phenylalanine, tyrosine, and tryptophan biosynthesis (Supplementary Data 5).

**Fig. 4:**
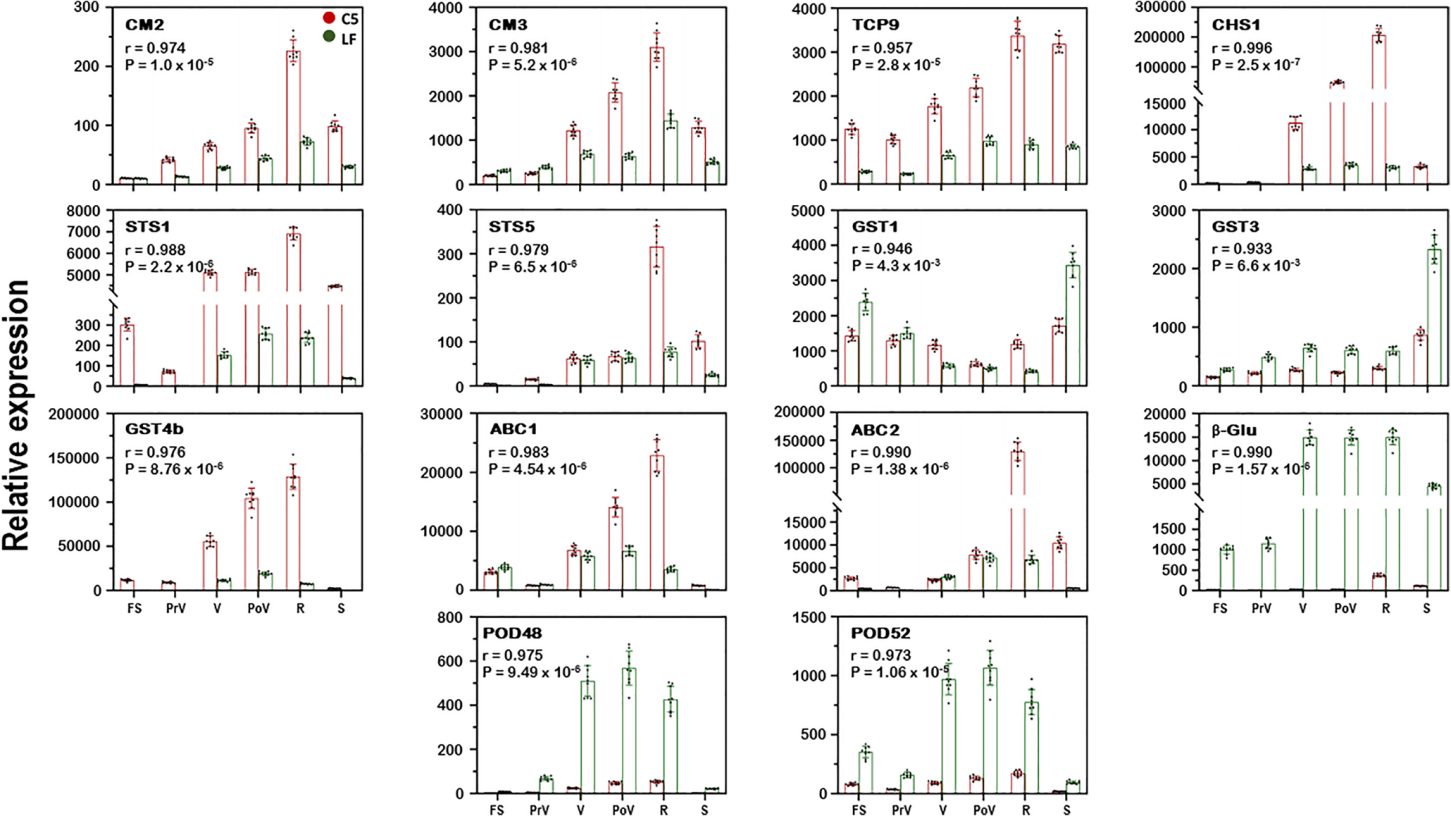
Comparison of expression profiles of 14 DEGs from module C3 as measured by qPCR in C5 and LF genotypes at different berry developmental stages. The genes encode chorismate mutase (*CM2* and *CM3*), stilbene synthase (*STS1* and *STS5*), teosinte branched1/cincinnata/proliferating cell factor (*TCP9*), chalcone synthase (*CHS1*), glutathione S-transferase (*GST1*, *GST3*, and *GST4b*), ATP-binding cassette transporters (*ABC1* and *ABC2*), β-glucosidase (*β-Glu*), and peroxidase (*POD48* and *POD52*). The *y*-axis represents the mean expression level (±SD) determined by qPCR. The *x*-axis in each chart represents the developmental stages. The mean expression level was calculated from three biological replicates each with three technical replicates (*n* = 9). Standard curves were used to calculate the number of target gene molecules per sample. These were then normalized relative to the expression of *Actin* and *EF1*. Correlations (*r*) between qPCR and RNA-seq expressions were calculated and their associated *P*-values are indicated.

Based on function annotation, a group of 14 hub genes found in the C3 module and highly associated with the measured traits was identified (Supplementary Figs. 11 and 12; Supplementary Data 8a). Interestingly, the correlation of these hub genes was compromised when WGCNA analysis was generated using the data of LF only or both (C5/LF) genotypes (Supplementary Figs. 12–14). The BP GO terms for glutathione metabolic process, chorismate metabolic process, and several terms related to cellular detoxification were significantly enriched in these hub genes, and the KEGG pathways for phenylpropanoid biosynthesis, flavonoid biosynthesis, glutathione metabolism, and phenylalanine, tyrosine, and tryptophan biosynthesis (Supplementary Data 9). The analysis of GO enrichments and KEGG pathways confirmed the effective WGCNA analysis of our multivariate and complicated data, as well as the narrow-down strategy^45,46^. The 14 genes were classified into three main groups (Fig. 4; Supplementary Fig. 15). Group-1 comprises two genes (*CM2/3*) involved in the shikimic pathway, encoding chorismate mutase that catalyzes the conversion of chorismate into prephenate and plays a key role in the biosynthesis of the essential aromatic amino acids tyrosine and phenylalanine^47^. Group-2 covers two genes encoding stilbene synthase (*STS1/5*), which belongs to the pterostilbene pathway. The STSs are characteristic of stilbene-producing plants and catalyze the biosynthesis of the stilbene backbone from three malonyl-CoA and one CoA-ester of a cinnamic acid derivative^48^. Group-3 includes the remaining 10 genes encoding proteins putatively associated with anthocyanin regulation (*TCP9*), biosynthesis (*CHS1*), transport (*GST1/3/4b* and *ABC1/2*), and degradation (*β-Glu* and *POD48/52*). Examining the WGCNA gene significance (GS) for evaluated traits, (i.e. correlation with the TAC and TPA) showed that *CHS1* has the highest GS with TACs (*r* = 0.99) and TPAs (*r* = –1.0). However, *GST1* and *ABC1* represent the lowest GS with TACs and TPAs, respectively (Supplementary Data 8a). Analysis of the 14 genes using qPCR demonstrated that the relative expression and the TPMs for each gene were significantly correlated (*r* > 0.97, *P* < 1.4 × 10^–5^), validating the expression measured by RNA-seq and suggesting that the 14 genes in module C3 may play a substantial role in anthocyanin accumulation (Fig. 4). This highlights that the co-gene network analysis using the WGCNA package, which has been used widely for similar analysis in other studies was meaningful in the biological sense in this study^49–51^. To validate the contribution of group-3 transcripts, their expression was assessed in Rosa berries that showed visible red color but with comparable TAC levels to those of unpigmented muscadines (Supplementary Fig. 16; Supplementary Table 2). Out of eight anthocyanin-related transcripts evaluated, six genes (*TCP9*, *CHS1*, *GST4b*, *ABC2*, *POD48*, and *POD52*) displayed an accumulation pattern associated with the level of TAC (*r* > 0.86, *P* < 3.7 × 10^–5^). However, the transcription of *ABC1* and *β-Glu* mRNAs did not show a significant correlation with the changes in anthocyanin levels but were more related to the emergence of pigments. These results suggested that the alteration of these anthocyanin-related genes would cause changes in anthocyanin accumulation.

In plants, genes involved in the flavonoid pathway function in concert to reach their ultimate levels^52^. The severity in phenotype between the muscadine genotypes used for transcriptome analysis should expose the majority, if not all, of anthocyanin regulation and biosynthesis structural genes involved in anthocyanins/PAs biosynthesis as DEGs^5,12^. *CHS1* played a positive central role in defining the final level of anthocyanin in colored muscadine berries. Excluding *CHS1*, five and three out of the 10 DEGs encode genes involved in anthocyanins/PAs transport and degradation, respectively. Despite the recently published results^31^, the transcriptome data suggested that all muscadines possess active anthocyanin regulatory and biosynthesis programs irrespective of berry color; however, the unpigmented phenotype in muscadine grapes might be due to a disorder event that occurred with anthocyanin transport and/or degradation pathways.

### Genome-wide association study for anthocyanin

**Fig. 5:**
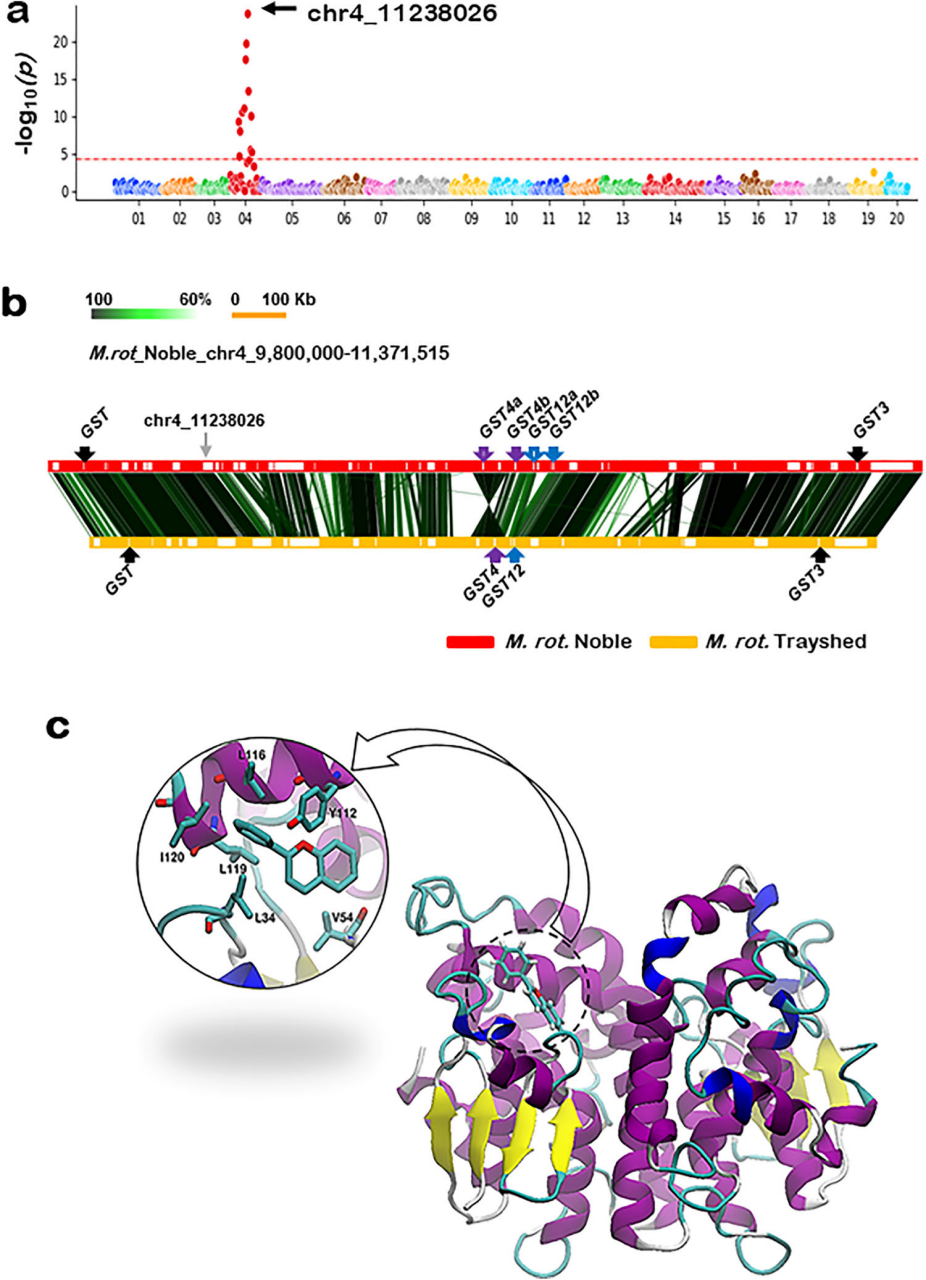
Genome analysis of *M. rotundifolia* berry color loci and modeling structure of GST4b protein underlying berry color diversity. **a** GWAS analysis of berry anthocyanin levels presented by the Manhattan plot. The *y*-axis indicates—log_10_(*p*) values of the result, and the *x*-axis refers to chromosome numbers. The Bonferroni-corrected *P*-value, 4.35, is presented by the dotted horizontal red line. The marker that shows the most significant—log_10_(*p*) value is indicated by a gray arrow with its name. **b** Comparative sequence analysis of the collinear regions of Noble and Trayshed genomes harboring the GSTs. The horizontal red and yellow bars indicate the sequences of Noble and Trayshed, respectively. The white squares in the two horizontal bars indicate the predicted genes. The vertical lines linking the two horizontal bars indicate similar regions and the similarity is depicted by color intensity. The location of GST homologs is indicated with arrows and names. In the Noble sequence, the GSTs absent in Trayshed are indicated by empty arrows, and the orthologous sets are indicated by the same color. **c** The 3-D modeling structure of anthocyanin–GST4b1 interaction based on the 5J4U template. The H-site is over-exposed with the specific amino acid interactions. The binding dominated by hydrophobic interactions with amino acid sidechains is shown.

A core set of 348 *M. rotundifolia* individuals were selected for GWAS analyses to determine genetic loci associated with anthocyanin accumulation. GWAS results revealed a strong peak on chromosome-4 (Fig. 5a). The biggest –log_10_*p* value appeared at the chr4_11238026 marker. To identify the genes around the marker, we investigated the coding genes between the flanking markers of the chr4_11238026 marker using the genome sequence of *M. rotundifolia* cv. Noble. Interestingly, the chr4_11238026 marker was recently identified by several reports as a major QTN in many muscadine berry color-related traits, including visible color, chroma, and luminosity^30–32^. Due to the black color of mature Noble berries, we expected the existence of functional genes associated with anthocyanin accumulation. The region contains 29 genes, including four genes that encode glutathione S-transferases (GST) involved in anthocyanin transport^53^ (Supplementary Data 10). To determine if there are additional GSTs, we investigated the genes in the extended flanking regions of the sequence. As a result, we could identify two more GSTs in the Noble muscadine genome (chr4_9,800,000 bp–11,371,515 bp; Fig. 5b). Comparing the sequence to the collinear region of the male *M. rotundifolia* cv. Trayshed genome sequence highlighted only four GSTs in the collinear region^54,55^. In the sequence comparison, GST and GST3 showed one copy of orthologs in both accessions; however, the GST4 and GST12 were duplicated in the Noble genome (GST4a/b and GST12a/b). Analysis of RNA-seq data among various tissues and berry developmental stages indicated that *GST12a/b* mRNAs are not expressed. Consequently, no further analysis was performed for these two genes.

### Identification of GST4 alleles among muscadine genotypes

To unravel the contribution of the GST4a/b to anthocyanin accumulation, the full-length genomic sequences of the two genes were isolated and sequenced from C5 and LF. PCR reaction with C5 was able to amplify the two genes; however, the LF genotype behaved like Trayshed and amplified only one ortholog. Based on nucleotide sequence identity, it was obvious that GST4b is the common gene between the two genotypes; however, GST4a was absent in LF. All GST4 genes have open-reading frames comprising three exons and two introns with the exon/intron boundaries occurring in the same positions (Supplementary Fig. 17a). Two different alleles were identified for GST4b (GST4b1/2) with three alterations in nucleotide composition that includes only one nonsynonymous polymorphism (C/T), leading to a predicted change in the highly conserved amino-acid residue Pro_171_ to Leu. Strikingly, the colored C5 genotype was heterozygous for GST4b; however, the unpigmented LF and the male Trayshed genotypes were homozygous for GST4b2 (Supplementary Fig. 17b). Given the nature of alleles’ distribution, we predicted that the allelic form with Pro_171_ would encode a functional GST and the allele with the substitution Leu_171_ should be inactive or at best differentially function, which agreed with the recently reported results^31^.

Assessment of TAC levels among the population suggested an average estimated at 240.8 ± 18.3 µg/g DW. The TAC trait considerably varied among the population with a wide range of 2328 µg/g DW. Among unpigmented genotypes, TAC levels ranged between 1.4-22.6 µg/g DW. Based on visual inspection, the colored berry genotypes were divided into two groups, red and purple/black with TAC levels of 10.1–58.3 and 61.4–2329.4 µg/g DW, respectively (Supplementary Data 1). These results suggested an obvious overlap in TAC levels between categories, particularly between green/bronze and red muscadines. HRM genotyping of GST4b suggested three allelic combinations of the C/T SNP marker at position 512 (C:C, C:T, and T:T; Supplementary Fig. 18; Supplementary Data 1). Out of 328 tested genotypes, all unpigmented muscadine berries (143 genotypes) exhibited a homozygous T-allele. Among colored genotypes, while only 15 genotypes were homozygous for C-allele, the majority of them (170 genotypes) hold the two different allele types (C:T). These data demonstrated the recessive inheritance of the loss-of-color berry trait. Interestingly, these results agreed with the recently reported data using KASP genotyping strategy to characterize muscadine populations^31^.

Statistical analysis between different groups was performed to determine the gene action and allele dosage effects. The analysis confirmed the significant differences in TAC levels (*P* = 1.3 × 10^−31^) between unpigmented/homozygote T-allele genotypes (4.8 ± 0.2 µg/g DW) and colored/C-allele holding genotypes (423.2 ± 25.4 µg/g DW). These results agreed with the recently published data using a subset of progeny from the “Supreme × Nesbitt” and “Black Beauty × Nesbitt” population^31^. To determine the effect of allele composition on color intensity, the analysis was performed between colored genotypes only, homozygote (723.0 ± 144.9 µg/g DW) and heterozygote (396.7 ± 23.7 µg/g DW) C-allele. Contrary to the recently reported findings^31^, the results demonstrated that the means of the TAC levels among colored muscadines significantly distinguish between homozygote and heterozygote genotypes (*P* = 3.3 × 10^−38^). These data suggested that the genetic variation in the GST4b sequence is the coordinator of color characteristics in muscadine grapes, including color emergence and intensity.

### GST4 protein structure

In plants, GSTs act as non-enzymatic carrier proteins (ligandins), which export specific endogenous compounds, including anthocyanins from the site of synthesis in the cytoplasm to vacuole^56^. The covalent glutathione (GSH-tag) mediates the recognition of molecules destined for vacuolar sequestration, resulting in GSH–anthocyanins conjugation. Subsequently, the conjugated anthocyanins will be transported, via the recognition of the GSH-tag molecule, by a tonoplast-localized ATP-binding cassette pump into the vacuole, the permanent storage compartment of anthocyanin^16,57^.

Molecular dynamics simulations were performed to examine the effect of the Pro_171_-to-Leu mutation. The muscadine GST4b1/2 shares a 74% identity with the poplar GST through which only muscadine GST4b1 and the poplar GST possess a proline residue at position 171. Docking simulation results indicated that the typical muscadine GST4b1 favorably binds GSH and different flavonoids, including anthocyanins, to the H-site region^58–60^ (Supplementary Table 3). The binding is dominated by hydrophobic interactions. The amino acid residue Tyr_112_ is likely involved in making pi–pi stacking interactions with the ligands, while the amino acids Leu_116_, Leu_119_, and Ile_120_ are involved in the hydrophobic interactions. However, the binding is favored by the presence of Val_54_ and Leu_34_ residues (Fig. 5c). The folded structure of GST4b2 displayed considerable changes within the H-binding site region. Due to the Pro_170_-to-Leu mutation, the binding site essentially becomes unfeasible for GSH and flavonoids (Supplementary Table 3). The structure has a small number of β-sheets and α-helices with the presence of a C-terminal entirely formed from α-helices. Accordingly, the mutation incident detected within GST4b2 made the protein inactive and unable to transport anthocyanins towards the vacuole.

In *Vitis* grapes, five GST genes have been studied in fruit^15,53^. Among them, VviGST3 and VviGST4 present the highest homology to *Arabidopsis* TT19. Nonetheless, by performing complementation studies in the *bz2* maize mutant, it was demonstrated that only VviGST1 and VviGST4 are involved in anthocyanin accumulation in the vacuole^53^. Based on the nature of the muscadine GST4b2 mutant, it seems that GST4 proteins are the only GSTs capable to transport anthocyanin molecules in muscadine.

### Enzymatic degradation of anthocyanin in muscadine

Anthocyanin quantification and transcriptome data suggested that all muscadines can produce anthocyanins at regulatory and biosynthesis levels. However, the mutation that occurred within the GST4b2 gene liable for anthocyanin transportation in unpigmented muscadines prevents the vacuolar accessibility of anthocyanins. In plant cells, the accurate delivery and sequestration of chemically reactive and potentially toxic metabolites pose a significant challenge for plant cells, which can simultaneously accumulate hundreds of different compounds^61^. Hence, the unpigmented muscadine genotypes should have an endogenous established mechanism to remove the anthocyanin accumulated and trapped within the cytoplasm compartment.

**Fig. 6:**
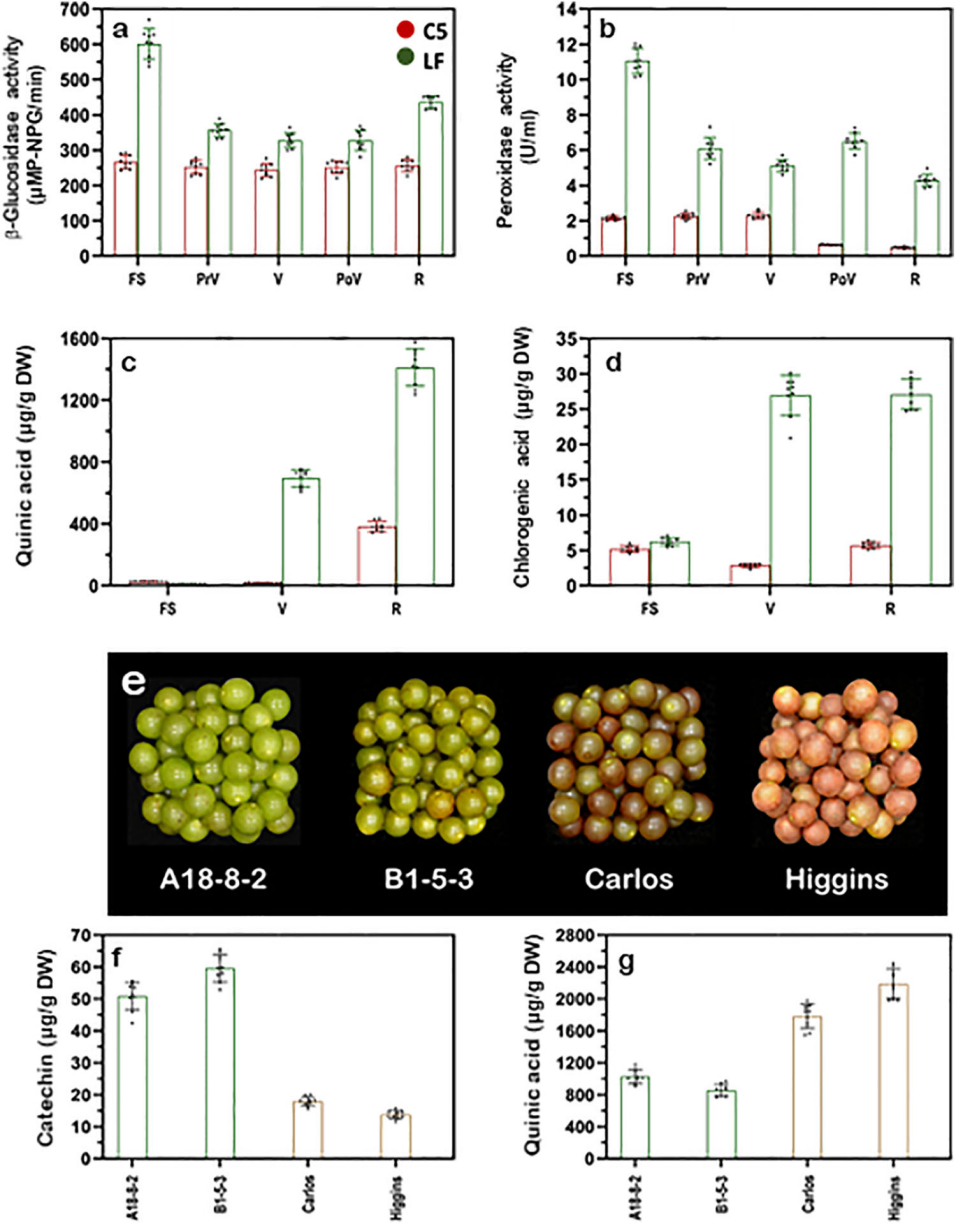
Enzymatic activities and metabolome quantification related to anthocyanin degradation in muscadine berry skin. Enzymatic activities of **a** β-glucosidase (uMP-NPG/min) and **b** peroxidase (U/ml) in muscadine berry skin during the development of C5 and LF genotypes. Levels of major quinones in muscadine skin, **c** quinic, and **d** chlorogenic acids (µg/g DW) during C5 and LF berry development. **e** A representative image for muscadine genotypes displayed green and bronze skin color at ripening. Levels of catechin (**f**) and quinic acid (**g**) (µg/g DW) at ripening for two green (A18-8-2 and B1-5-3) and two bronze (Carlos and Higgins) muscadine skin color. Bars are the mean of three biological and technical replicates (*n* = 9; ±SD).

Earlier investigations demonstrated that anthocyanin degradation might involve active enzymatic degradation or non-enzymatic chemical degradation^62,63^. Quite a few candidate enzyme families have been reportedly contributing to anthocyanin degradation in planta, including β-glucosidases, polyphenol oxidases (PPOs), and peroxidases (PODs)^64–66^. However, several lines of evidence support the specific involvement of β-glucosidase and POD enzymes in the current study. The cytosolic β-glucosidase hydrolyzes the anthocyanin molecules via stripping their glucose residues, generating the colorless PAs^67^. Interestingly, PAs abundantly accumulated during LF berry development; however, their accumulation declined with development in C5 (Fig. 1c, e, g). Two enzymes, leucoanthocyanidin reductase (LAR) and anthocyanidin reductase (ANR), can produce the PAs polymers^5^. Yet, these two genes could not be considered as limiting factors for PAs accumulation in this study since they have not arisen as DEGs in the transcriptome data. Nevertheless, the abundance of the *β-Glu* mRNA in LF berry during development (~8.4-fold>in C5; Fig. 4; Supplementary Fig. 15) suggested the involvement of a stimulated β-glucosidase catabolic pathway in PAs accumulation. Assessing the β-glucosidase activity during LF/C5 berry development confirmed the enzyme contribution (~1.6-fold>in C5; Fig. 6a). The β-glucosidase activity in LF seems to be coordinated in genotype- and developmental stage-dependent manners, which differed from C5 that exhibited constant moderate activity. Apparently, the synthesized anthocyanins are trapped in the cytosol compartment of unpigmented muscadines without the potential ability to move into the vacuole. To overcome the absence of active anthocyanin-GST transporter, the cell stimulates hydrolysis reaction, converting anthocyanins into another GST-transportable form, PAs. Consequently, the accumulated PAs should be transported to the vacuole via several accessible PAs-GSTs (e.g., *GST1* and *GST3*; Fig. 4).

Further, anthocyanins/PAs can be oxidized by PPOs and/or PODs pathways in-planta, resulting in an accelerated degradation process via a coupled oxidative reaction^64–66,68^. An earlier study showed that PPOs activity is the main factor underlying anthocyanin degradation in red muscadine grape juice^69^. Assessment of PPOs activity during C5/LF berry development revealed an activity that was not consistent with anthocyanin accumulation, excluding any potential PPOs involvement (Supplementary Fig. 19). Nonetheless, several lines of evidence suggested the direct contribution of PODs activity to the degradation of PAs in LF muscadine. First, the abundance of the *POD48* and *POD52* transcripts in LF berry during development were ~3.4-fold and ~2.5-fold higher than in C5, respectively (Fig. 4). Further, the assessment of PODs activity during C5/LF berry development showed that both genotypes exhibited a similar impaired pattern along with development, but with ~4.1-fold higher levels in LF (Fig. 6b).

The direct oxidation of anthocyanins/PAs via PODs led to the accumulation of the metabolites involved in the browning pathway (i.e., quinones)^64,70^. HPLC quantification of major quinones accumulated in muscadine skin displayed elevated levels of quinic (~4.9-fold) and chlorogenic acids (~4.4-fold) in LF skin relative to C5, particularly at V and R stages (Fig. 6c, d). Accordingly, PODs are most likely the candidate for the oxidizing reaction than PPOs. Several studies revealed that PODs are the likely candidates mediating the in-planta degradation of anthocyanins because they are located in vacuoles, whereas PPOs are localized to plastids^71–73^. These results strengthen the notion that β-glucosidases and PODs are the major pathways underlying anthocyanin degradation within the muscadine berry context. On the other side, the significant anthocyanin degradation activity detected in colored grapes, including C5 should not result in a dramatic change in visible color, as the pigments continue to be synthesized in parallel to their catabolism^74^.

Visual inspection of the muscadine population indicated that some unpigmented genotypes develop bronze color at ripening; however, others remain green. Considering that all unpigmented genotypes exhibit higher degradation activity of anthocyanins, we presumed that the bronze color is developed due to the stimulated oxidative reaction of PAs, resulting in the accumulation of quinones; the precursor of browning compounds^64,70^. To test this hypothesis, the major PAs (catechin) and quinones (quinic acid) in muscadine were quantified in the skin of two green and bronze genotypes (Fig. 6e). Interestingly, the level of catechin detected in green genotypes was ~2.8 to ~4.2 times higher than in bronze genotypes (Fig. 6f). By contrast, the level of quinic acid detected in bronze grapes was ~1.7 to ~2.6 times higher than in green genotypes (Fig. 6g). Apparently, unpigmented muscadines exhibit high PAs accumulation; however, it seems that the ultimate visible color of green with higher PAs or bronze with higher quinones depends on the PODs activity rate. Hence, the difference between green and bronze genotypes should be due to varied PODs activity. However, it is necessary to test more green and bronze individuals to set up the mechanism. These results suggested that anthocyanins degradation might be a stretchy process but completely controlled to attain the maximal benefit for plants^2–4^.

Plants can accommodate a wide variety of unexpected events, including mutations. Plasticity plays important role in ecosystems, agriculture, and landscape esthetics. The colored muscadines hold at least one active GST4b1 allelic form that can transport anthocyanins successfully into the vacuole; however, the unpigmented muscadines carry only the inactive GST4b2 allele. Despite the recent discovery of GST4b as a limiting factor for the color trait in muscadine^31^, it was not clear why the mutation in GST4b2 encoded an inactive protein. Through the molecular dynamics simulations analysis, we predicted that the mutation caused a considerable modification in the GSH/flavonoids binding site, making the protein unfeasible for anthocyanins transportation. Gene knockout and complementation studies have established that GSTs are indelibly involved in anthocyanin transport. A maize knockout mutant of a single GST (*bz2*) produces bronze skin kernels due to the disabled transport of anthocyanins into vacuole^17^. The transient expression of active *Bz2* or a petunia GST (*An9*) in a *bz2*-deficient mutant was able to complement the deficiency, resulting in kernels with red spots^14,16^. Unpigmented muscadine genotypes behaved similarly to the *bz2*-deficient mutant in terms of holding a single inactive GST protein, which gives rise to bronze/green fruit.

Under such circumstances, plants can rapidly respond to sudden stimuli by rebuilding their system biology architecture to modify whole plant strategies, avoiding the impact of maintaining productivity. The fundamental importance of these processes has prompted considerable research into how plants reacted to the changes in their growth behavior. Accordingly, we showed the mechanism by how the plant responds to the mutation stimulus by biochemically manipulating the anthocyanins trapped in the cytoplasm to another GST transportable form, PAs. Several studies suggested the anthocyanin catabolism pathway, as a potential strategy that can coordinate the plant’s behavior and permit flexible and appropriate modulation of anthocyanin levels^62–64,66,73^. The transcriptome data, enzymatic activity, and quantification of applicable metabolome supported a stimulated catabolism procedure in unpigmented muscadine. If this is the case, the over-accumulation of anthocyanins in the cytoplasm will likely turn on an alert signal via stimulating the degradation pathways, clearing out anthocyanins trapped within the cytoplasm compartment.

Considerable effort has been invested in clarifying the mechanisms underlying fruit pigmentation, including muscadine grape. In plants, three major associated pathways have been identified to be involved in anthocyanin accumulation, including regulatory, biosynthetic, and transportation^9,12,15^. Initially, the authors proposed a potential negative feedback mechanism strategy, resulting in a shutdown of the anthocyanin biosynthesis pathway to avoid excessive anthocyanins accumulation within the cytoplasm compartment^75^. However, the transcriptome data did not support such a procedure, but the contrary suggested comparably active regulatory and biosynthesis processes between colored and unpigmented genotypes. However, the characterization of the color trait in muscadine was able to identify a major berry color locus on linkage group-4^30,32^. The recently published report and the current study established that the distinguishable color/unpigmented phenotypes are due to a mutation that occurred within the GST4b2 gene, highlighting the dominant inheritance of color trait^31^.

**Fig. 7:**
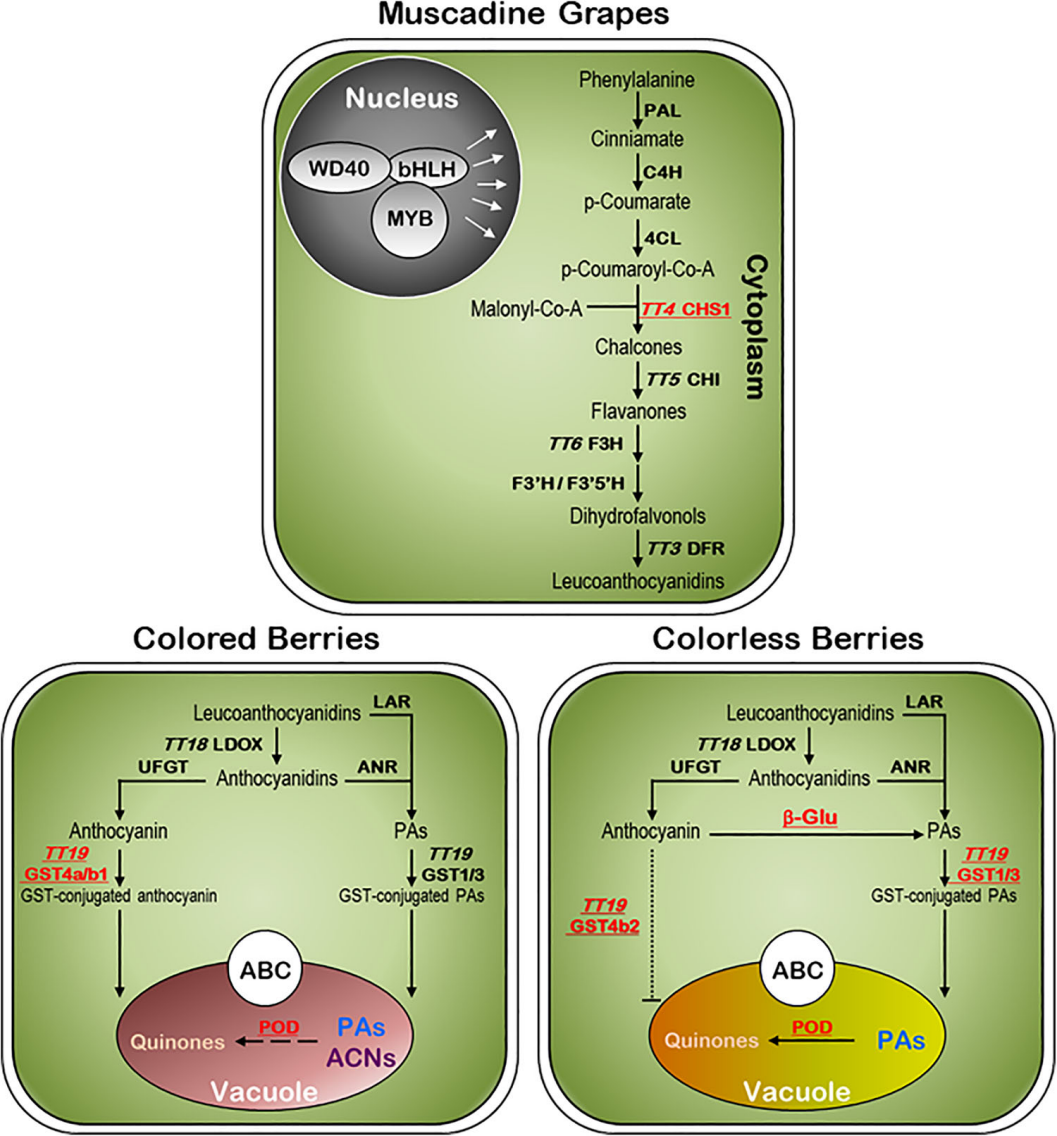
Diagram of the anthocyanin pathway assigned with different structural genes. The CHS1 gene was more associated with color emergence and intensity. The major difference between colored and unpigmented muscadine genotypes appears at a late stage associated with anthocyanin transportation. The active GST4a/b1 and ABC1/2 mediate anthocyanin/GSH co-transport in colored muscadines. The presence of only the inactive GST4b2 in unpigmented muscadines prevents anthocyanin transport to the vacuole. The stimulated β-glucosidase activity hydrolyzes anthocyanins into PAs that can be transported to the vacuole via GST1 and GST3. In all muscadines, the stimulated POD activity oxidizes the anthocyanins (ACNs) and PAs, resulting in the accumulation of quinones with considerable more activity in unpigmented muscadines.

In conclusion, we designed the next scenario for anthocyanin accumulation in muscadine grapes (Fig. 7). All muscadine grapes can produce anthocyanin due to active regulatory and biosynthesis pathways. However, the CHS1 enzyme is the critical limiting step in coordinating anthocyanins accumulation in colored genotypes through which the higher CHS1 activity is associated with high anthocyanin levels. In colored muscadines, the anthocyanins are typically transported from the site of synthesis in the cytoplasm to the site of permanent storage in the vacuole via GST4a/4b1 and ABC1/2 proteins and, consequently, the pigments could be visualized. In unpigmented muscadines, the synthesized anthocyanins remain potentially trapped in the cytoplasm due to the availability of only the defected GST4b2 protein. To avoid the accumulation of anthocyanins in the cytoplasm, the cell stimulated the anthocyanins degradation program. The activated β-glucosidases enzyme converts the anthocyanins into the colorless PAs within the cytoplasm compartment. Consequently, the PAs should be transported to the vacuole via GST1 and GST3. Within the vacuole compartment, all muscadines degrade the anthocyanins/PAs, a process that is more stimulated in unpigmented muscadines. The degradation occurs via PODs activity, converting the anthocyanins/PAs into quinones, the precursors of browning compounds, resulting in a bronze color. This pattern is more visible in unpigmented muscadines due to higher PODs activity and the absence of colored pigments.

## Methods

### Chemicals

Standards of delphinidin-3-O-β-glucopyranoside chloride (dp-3-glc), cyanidin-3-O-β- glucopyranoside chloride (cy-3-glc), petunidin-3-O-β-glucopyranoside chloride (pt-3-glc), peonidin-3-O-β-glucopyranoside chloride (pn-3-glc), malvidin-3-O-β-glucopyranoside chloride (mv-3-gl), (−)-epicatechin, (−)-epicatechin gallate, (−)-epigallocatechin, (+)-catechin, procyanidin dimers B1, procyanidin dimers C1, quinic acid, and chlorogenic acid were purchased from (Sigma-Aldrich, St. Louis, MO, USA). The *p*-nitrophenyl *p*-d-glucopyranoside (*p*NPG), and vanillin were purchased from (Sigma-Aldrich, St. Louis, MO, USA). HPLC grades of acetonitrile, methanol, and formic acid were purchased from (VWR-USA). Anthocyanins Assay Kit (Cosmo Bio, Carlsbad, CA, USA).

### Standards preparation for HPLC

Stock standard solutions (100 μg/ml) of the anthocyanins dp-3-glc, cy-3-glc, pt-3-glc, pn-3-glc, and mv-3-gl were prepared with 1% HCl in methanol and stored for a week at –20 °C. Each week new stock solutions were designed to ensure the freshness of the standards. Working standard solutions of 100, 75, 50, and 25 μg/ml were prepared to build the calibration curve for each compound. The working standards were prepared daily to check the method’s performance and possible degradation of the stock solutions. Standard solutions for proanthocyanidins were prepared for each procyanidin oligomer class at different concentration levels (5–2000 μg/ml) in methanol for building standard curves.

### Plant materials

Berry samples were collected at the ripening stage from 5-years old muscadine grapevine (*Muscadine rotundifolia*) genotypes, including nine standard cultivars Scuppernong, Pam, Granny Val, Carlos, Late Fry (LF), Rosa, Farrer, Floriana, Noble, and one breeding line C5-9-1 (C5—“Ison × Fry”) grown at the experimental vineyard of the Florida A&M University (Tallahassee, FL, USA). The C5 and LF were selected for further analysis according to their diversity in berry color characteristics, black, and bronze, respectively. Berry samples from assigned genotypes were collected at different developmental stages, including fruit-set (FS), pre-véraison (PrV), véraison (V), post-véraison (PoV), and ripening. The berries were separated into two tissues at the ripening stage: ripe-skin (R) and ripe-seed (S). Five clusters/replicate and three replicates/genotype were randomly collected for all developmental stages. All samples were immediately frozen in liquid nitrogen and stored at −80 °C for further analysis.

### Berry skin color measurement

The skin color was measured at four different positions around the equator of the berries. Color coordinates (L*, a*, and b*) were determined by a Konica Minolta, CR-10 Plus fruit colorimeter (Konica Minolta, Inc. Chiyoda City, Tokyo, Japan) used for a deeper unbiased color index. The instrument was calibrated with a white blank calibration tile before each measurement. Luminance coordinate L* means the luminosity from 0 (black) to 100 (white). The chromaticity value a* means red when positive and green when negative. The chromaticity coordinate b* means yellow when positive and blue when negative. The chroma (*C*) and hue angle (*H*) values were calculated from a* and b* values, with *C* calculated as (*a*^2^ + *b*^2^)^1/2^. For the *H* angle, the calculation depended on the obtained charge of a* and b* values. If the a* and b* values were positive, the *H* = Arc Tan (*b*/*a*). If the a* value was positive and the b* value was negative, the *H* = 360 + Arc Tan (*b*/*a*). However, if the a* value was negative and the b* was positive, or both values were negative, the *H* = 180 + Arc Tan (*b*/*a*). All color parameters were generated from three independent berries.

### Anthocyanin quantification

Lyophilized materials were finely ground, and ~12 g of powder tissues were homogenized in 100 ml of methanol (1% HCl). All extractions were performed by shaking (150 rpm) for 24 h/20 °C in the dark. All extracts were filtered, supernatants were dehydrated, and dried extracts were stored at 4 °C in the dark. The total anthocyanin content (TAC) was assessed using Anthocyanins Assay Kit (Cosmo Bio, Carlsbad, CA, USA) with minor modifications to accommodate the reaction in 96-well microplates^33^. Briefly, the stock solution of skin extracts was prepared at 10 mg/ml in DMSO for the TAC assay. The reaction composition and steps were as follows: in two side-by-side wells, a volume of 200 µL of Reagent-A (KCl—25 mM) and 200 µL of Reagent-B (Na Acetate—0.4 M, pH 4.5) was added. Then, 20 µl of extract solution was added to each well. The reactions were mixed by slow shaking for 1 min and incubated in the dark for 10 min at room temperature. The absorbance measurements were performed at *λ* = 510 nm (maximum anthocyanin absorption) and *λ* = 700 nm (for turbidity correction) using the ACCURIS SMART Plate Reader spectrophotometer (Thomas Scientific, Swedesboro, NJ, USA). TAC estimation was generated from three biological replicates, and each sample was run in three technical replicates and expressed as a microgram of delphinidin equivalents per gram dry weight (µg/g DW). HPLC method was used for individual anthocyanin content (IAC) quantification^76^. A 20 μL aliquot of the purified extract was injected into Shimadzu-HPLC equipped with a diode array detector (Fluorescence Detector, Shimadzu, Japan). The quantification was performed at *λ*_ex_ = 290 nm and *λ*_em_ = 530 nm with Agilent Pursuit 5 column (C18 ODS 10.0 × 250 mm). The mobile phase was composed of 3:57:40 (v/v) formic acid–water–methanol solution. The column flow rate was 0.8 ml/min with a running time of 35 min at 40 °C. Anthocyanins were identified by comparing the retention time with standards (Supplementary Fig. 20a; Supplementary Table 4). The data were obtained from three biological and technical replicates and expressed as a microgram of delphinidin equivalents per gram of dry weight (µg/g DW).

### Proanthocyanidin quantification

For total proanthocyanidins (TPAs) extraction, a 200 mg tissue powder was subjected to two-step extraction with 10 ml of solvent A (first step): acetone/water (80:20; V:V) followed by extraction with 10 ml of solvent B (second step): methanol/water (60:40; V:V). For each extraction step, samples were sonicated for 20 min, centrifuged (10,000 rpm/10 min/4 °C), and the supernatant was collected^77^. The normal phase HPLC method was used for individual proanthocyanidins (IPAs) determination^78^. Separation was performed on an Agilent Pursuit 5 column and monitored by fluorescence detection at *λ*_ex_ = 276 nm and *λ*_em_ = 316 nm. An external standard consisting of commercially available monomeric and dimeric procyanidins was used for quantification (Supplementary Fig. 20b; Supplementary Table 5). The data were obtained from three biological and technical replicates and expressed as a microgram of catechin equivalents per gram of dry weight (µg/g DW).

### The *β*-glucosidase and peroxidase activity assays

The crude enzyme extract was prepared by adding 10 ml of potassium phosphate buffer (50 mM, pH 7.0) to one gram of frozen berry skin powder. The mixture was kept on ice for 30 min with vortex every 10 min. After double extraction, the supernatant was collected by centrifugation (10,000 × *g*/10 min at 4 °C). The hydrolytic activity of β-glucosidase was determined by measuring the release of *p*-nitrophenol from *p*-nitrophenyl *p*-d-glucopyranoside (*p*NPG). All samples were assayed at 50 °C in potassium phosphate buffer (KPB, 50 mM, pH 7). The *p*NPG (15 µl, 100 mM dissolved in DMSO) and enzyme sample (100 µl) were added to a 2.9 ml potassium phosphate buffer. After mixing, the absorbances were recorded every 30 s for a total of 120 s. Instead of the enzyme sample, 100 µl buffer solution was added for determining the spontaneous hydrolysis of *p*NPG, which was little within the time of determination. The enzyme activity was calculated by subtraction of the spontaneous hydrolysis of the *p*NPG. One unit of β-glucosidase hydrolysis activity was defined as the amount of enzyme that releases 1.0 µmol *p*-nitrophenol per minute under such conditions^79^. Peroxidase enzyme activity was assessed in skin extracts using the Peroxidase Activity Assay kit (Elabscience, Houston, TX), according to the manufacturer’s instructions. The data were obtained from three biological and technical replicates (*n* = 9).

### RNA extraction and RNA-seq library construction

The total RNA from the muscadine berry tissues was extracted using the CTAB protocol. A total of 18 RNA-seq libraries (three biological replicates at three stages of FS, V, and R from the C5 and LF muscadine genotypes) were constructed using NEBNext Ultra II RNA Library Prep Kit for Illumina (New England Biolabs, Ipswich, MA). These libraries were multiplexed in an equal amount for paired-end 150-base sequencing in two lanes of NovaSeq 6000 (Illumina, San Diego, CA) at the Novogene Co., Ltd (Sacramento, CA).

### RNA-seq data preprocessing and identification of differentially expressed genes

The Illumina sequencing of the multiplexed RNA-seq libraries yielded 18 FASTQ files of sequences. The reads quality was checked by FASTQ (https://www.bioinformatics.babraham.ac.uk/projects/fastqc/) before and after trimming using Trimmomatic v0.39^80^. Trimmed reads were then subjected to Salmon in non-alignment-based mode to estimate transcript quantification^81^. All samples were mapped to the de novo muscadine transcriptome^32^. Differentially expressed genes (DEGs) during berry development were identified between consecutive developmental stages (V-FS and R-V) within the genotype and between equivalent stages of C5 and LF, using DESeq2 and EdgeR package setting on default parameters^40,41^. Common and unique DEGs of each comparison generated by DESeq2 and EdgeR pipelines (DEGs, *P*_FDR_ < 0.05, log2fold change > 1.5 or <–1.5) were considered to be expressed. The annotation information of DEGs was identified based on the reference genome annotation of muscadine grape^32^. Finally, the web-based tool Venny was used to construct the consensus result^82^.

### Weighted gene co-expression network analysis

Co-expression network modules were constructed using the variance stabilizing transformed values of RNA-seq data and the R package WGCNA (v1.69)^43^. Barely expressed genes were removed by DESeq2 and the remaining genes in C5 were used in module construction. Additionally, WGCNA module construction based on LF only or both C5 and LF genes was generated. For C5 data, the co-expression modules were obtained using the default settings, except that the soft threshold power was 10, TOMType was engaged, minModuleSize was 30, mergeCutHeight was 0.25, and scale-free topology fit index 0.8 (*r* = 0.8). A module eigengene (ME) value, which summarizes the expression profile of a given module as the first principal component, was calculated and used to evaluate the association of modules with anthocyanin property, including the TAC and IAC traits from C5 at different developmental stages. Furthermore, the number of non-redundant DEGs from C5_stages_, LF_stages_, and C5_stage_–LF_stage_ comparisons were assigned.

### GO enrichment and KEGG pathway analyses

GO and KEGG enrichment analyses were assigned using the g: Profiler website by applying Benjamini–Hochberg multiple testing correction methods with *P*_FDR_ < 0.05^44^. However, since the gene ID of the muscadine transcriptome is not supported, the Ensembl gene ID of *Vitis vinifera* was used instead, although some genes do not have such *V. vinifera* ID. The Cytoscape plug-in ClueGO was used to visualize the non-redundant BP terms and KEGG pathways^83^.

### Validation of DEGs subsets by quantitative PCR analysis

The DNase-treated RNA (4 µg) was reverse transcribed in a reaction of 50 µl using the High Capacity cDNA Reverse Transcription Kit (Applied Biosystems, Foster City, CA, USA). The gene-specific primers were designed using Primer Express (v3.0, Applied Biosystems, Foster City, CA, USA) (Supplementary Data 8a). The real-time quantitative PCR (qPCR) assays were performed using 20 ng of cDNA and 300 nM of each primer in a 10 µl reaction volume with SsoAdvanced Universal SYBR Green Supermix (Bio-Rad Laboratories, Hercules, CA, USA). Three biological and three technical replicates for each reaction (*n* = 9) were analyzed on a CFX384 Touch Real-Time PCR Detection System instrument (Bio-Rad Laboratories, Hercules, CA, USA) with the first step of 95 °C for 5 min followed by 40 cycles of 95 °C for 10 s, 60 °C for 10 s, and 72 °C for 20 s. Melting curves were generated using the following program: 95 °C for 15 s, 60 °C for 15 s, and 95 °C for 15 s. Transcript abundance was quantified using standard curves for the target and reference genes, generated from serial dilutions of PCR products from corresponding cDNAs. The transcript abundance was normalized to the reference genes *MrActin* and *MrEF1*, which showed high stability across the different muscadine genotypes and tissues used in this study. The geometric mean of the selected housekeeping genes was validated as an accurate normalization factor. The data were presented as an average of nine replicates (±SD).

### Genotyping, phenotyping, and GWAS analysis

The genotyping data and procedure from the previously characterized muscadine population were used in the current study^32^. However, the phenotyping of the anthocyanin trait was performed via assessing TAC in ripe berries. For GWAS analysis, we used the imputed genotypes and the subpopulation number *k* = 15, which were previously generated. The GWAS analysis was performed by MLM (Q + K) workflow implemented in TASSEL v5.2.79 software^84^. A Bonferroni-corrected *p*-value of 0.05 was calculated with 1138 markers, resulting in a value of 4.39 × 10^−5^. The markers significantly associated with the trait were determined using a threshold of –log_10_(4.39 × 10^−5^), which was calculated as 4.35. Therefore, the markers with—log_10_(*p*) > 4.35 were considered to show a significant association with the analyzed phenotype. Manhattan plot of the –log_10_(*p*) values for each marker in the muscadine chromosome sequences was generated with an in-house Python script.

### High-resolution melting analysis

HRM analysis was carried out on a LightScanner HR384 (BioFire, Salt Lake City, UT) using 328 muscadine genotypes (56 cultivars and 272 breeding lines) and primers listed in Supplementary Data 8b^85,86^. Briefly, DNA was quantified using PicoGreen (Thermo Fisher, Waltham, MA) and normalized to a concentration of 5 ng/μL. HRM was performed at a ramp of 10 °C in an appropriate temperature range with 0.1 °C increments every 2 s. The fluorescent data were acquired at each of the HRM steps subjected to automatic gain optimization. The melting data were normalized by adjusting the start and end fluorescence signals of all samples to the same levels. HRM curve for each individual was visually scored. Different genotypes were identified by examining both normalized, difference, and derivative melt plots. The Student’s *t*-test was carried out to test the statistical significance of the data.

### Protein structure prediction

The structure *Populus trichocarpa* GST–flavonoids complex (PDB ID: 5J4U) was used as a template to build a homology model of muscadine GST4b proteins by the MODELLER package. The resultant structures were optimized using the generalized-born model for the solvent of AMBER/16 suite of Molecular Dynamics algorithms. A 15,000 steps minimization was performed, followed by a 5 ns molecular dynamics simulation to slowly heat the structure to 300 K, 10 ns of equilibration at 300 K, and 50 ns of the production run molecular dynamics at 300 K. The generalized-born algorithm was used for implicit solvent throughout the simulations. The average structure of the final 20 ps of the production run was used for the docking simulations after a 15,000-step minimization to remove any clashes in the structure. The LeDock software was used to conduct the docking simulations of both GSH and flavonoids. The box was set to be centered on the H-binding site.

### Statistics and reproducibility

For any biological study analysis (i.e., metabolites quantification, enzymatic activity, and qPCR assays), the experiment was conducted using three biological and technical replicates and the data were presented as an average of nine replicates (*n* = 9; ±SD). The RNA-seq data were generated from three biological replicates (*n* = 3; ±SD). The RNA-seq data were analyzed using two different pipelines, Edge R and DESeq2, to validate the analysis.

For GWAS analysis, the population was represented by 348 individual muscadine genotypes. For the HRM analysis, the study was conducted using 328 individual muscadine genotypes. The population used for genomic studies was carefully selected to exhibit sufficient diversity, validating the related studies. The data related to population characterization for anthocyanin levels were assessed for 3 consecutive years. All muscadine vines included in this study were represented by three copy vines that are located in different places within the vineyard through which each vine represents a biological replicate. Samples exhibiting similar developmental stages (age) from different locations within the same vine were collected (technical replicate). This strategy allowed us to ensure the reproductively of the results, irrespective of the potential involvement of environmental and management factors.

### Reporting summary

Further information on research design is available in the Nature Research Reporting Summary linked to this article.

## Supplementary information

Supplementary Information

Description of Additional Supplementary Files

Supplementary Data 1

Supplementary Data 2

Supplementary Data 3

Supplementary Data 4

Supplementary Data 5

Supplementary Data 6

Supplementary Data 7

Supplementary Data 8

Supplementary Data 9

Supplementary Data 10

Reporting Summary

## Data Availability

All RNA-seq data generated during the current study are available in the NCBI GenBank: PRJNA775666 and PRJNA810835.
