# Supplementary material for: A recessive mutation in muscadine grapes causes berry color-loss without influencing anthocyanin pathway
Source: Commun Biol. 2022 Sep 24;5:1012. doi: 10.1038/s42003-022-04001-8 (PMC9509324; doi:10.1038/s42003-022-04001-8)
Supplement: Supplementary file 1 — Supplementary Information [file 42003_2022_4001_MOESM1_ESM.pdf]

Supplementary Information

A recessive mutation in muscadine grapes causes berry color-loss without influencing anthocyanin pathway

Ahmed Ismail[1,2†], Pranavkumar Gajjar[1†], Minkyu Park[1], Abdulla Mahboob[4], Violeta Tsolova[1], Jayasankar Subramanian[5], Ahmed G. Darwish[1,3]*, Islam El-Sharkawy[1]*

This file includes:

- Supplementary Tables.
- Supplementary Figures.

Supplementary Tables

Supplementary Table 1. Colorimetric parameters of muscadine grape berry skin genotypes.

Genotype	Color	L*	a*	b*	Hue	Chroma
**Scuppernong**	Bronze	36.9±1.1	12.1±1.5	13.2±1.3	16.9±1.7	17.9±1.8
**Pam**	Bronze	41.8±1.7	1.2±0.2	14.6±1.1	38.5±0.9	14.6±1.1
**Granny Val**	Bronze	38.1±0.8	1.9±0.1	14.9±0.5	37.3±0.3	15.1±0.5
**Carlos**	Bronze	42.8±2.1	4.5±1.1	15.2±1.4	31.3±2.6	15.8±1.3
**Late Fry**	Bronze	40.5±0.2	7.6±0.3	18.6±0.7	28.7±1	20.1±0.6
**Rosa**	Red	23.6±0.3	7.6±1	1.9±0.1	354.6±1.6	7.9±0.9
**Farrer**	Dark red	20.4±0.4	2.5±0.3	-0.2±0.1	340±2.6	2.5±0.3
**Floriana**	Black	21.9±0.2	1.0±0.1	0.2±0.1	352.6±2.6	1.0±0.1
**Noble**	Black	20.8±1.5	1.8±0.8	0.5±0.3	357.9±5.3	1.9±0.8
**C5-9-1**	Black	18.8±1.2	2.0±0.3	0.2±0.2	350.1±4.5	2.0±0.3

Data were obtained from ripe berries of corresponding genotypes. Values are expressed as mean ± SD (n=3).

Supplementary Table 2. Total and Individual anthocyanin content in the extracts of muscadine grape genotypes.

Genotypes	Total anthocyanin content (µg/g DW)					
	**TAC**	**Delphinidin**	**Cyanidin**	**Petunidin**	**Peonidin**	**Malvidin**
**Scuppernong**	5.4 ±1.4	0.7 ±0.3	3.7 ±1.1	0.7 ±0.2	0.2 ±0.2	0.2 ±0.2
**Pam**	3.9 ±0.9	0.2 ±0.1	3.6 ±0.7	0.1 ±0	0.1 ±0	0.1 ±0
**Granny Val**	5.8 ±1.0	1.0 ±0.4	4.7 ±0.8	ND	ND	ND
**Carlos**	5.4 ±1.3	1.3 ±0.4	3.9 ±0.6	ND	ND	ND
**Late Fry**	4.7 ±2.3	1.8 ±0.9	2.2 ±1.2	0.2 ±0.1	0.4 ±0.2	ND
**Rosa**	11.7 ±2.3	2.0 ±0.7	8.0 ±1.4	1.2 ±0.3	0.3 ±0	0.5 ±0.2
**Farrer**	225.5 ±18.0	66.5 ±4.0	71.5 ±5.0	72.5 ±12.5	3.0 ±0.5	17.5 ±2.0
**Floriana**	1199.1 ±40.8	860.2 ±30.6	81.6 ±9.1	208.5 ±13.6	12.5 ±2.3	43.1 ±3.4
**Noble**	2329.4 ±106.8	1346.9 ±83.2	81.8 ±12.2	534.2 ±61.5	297.5 ±23.0	68.3 ±10.1
**C5-9-1**	496.1 ±23.5	319.8 ±9.4	116.1 ±14.1	42.9 ±2.4	3.2 ±0.2	14.1 ±1.5

Extracts were performed using ripe berries of corresponding genotypes. Values are expressed as mean ± SD (n=9), ND: not detected.

Supplementary Table 3. Predicted binding free energies of HSH and flavonoids with the different glutathione S-transferase versions.

**Ligand**	**GST4b1**	**GST4b2**
GSH	–4.94	0.73
Flavylium	–1.20	1.45
Cyanidin	–1.86	2.88
Epicatechin	–2.19	2.48
Kaempferol	–0.32	4.26
Quercetin	–1.48	2.87

Supplementary Table 4. Identification of anthocyanins in muscadine skin.

Peak	Retention Time t_R (min)	Anthocyanin
**1**	11.08	Delphinidin-3,5-diglucoside
**2**	13.07	Cyanidin-3,5-diglucoside
**3**	16.34	Petunidin-3,5-diglucoside
**4**	18.19	Peonidin-3,5-diglucoside
**5**	19.54	Malvidin-3,5-diglucoside

Supplementary Table 5. Identification of PAs in muscadine skin.

Peak	Retention Time t_R (min)	Proanthocyanidin
**1**	17.7	Epigallocatechin cinnamate
**2**	20.4	(+)-Catechin
**3**	24.9	Procyanidin B1
**4**	28.9	(-)-Epicatechin gallate
**5**	34.3	Procyanidin C1

<u>Supplementary Figures</u>

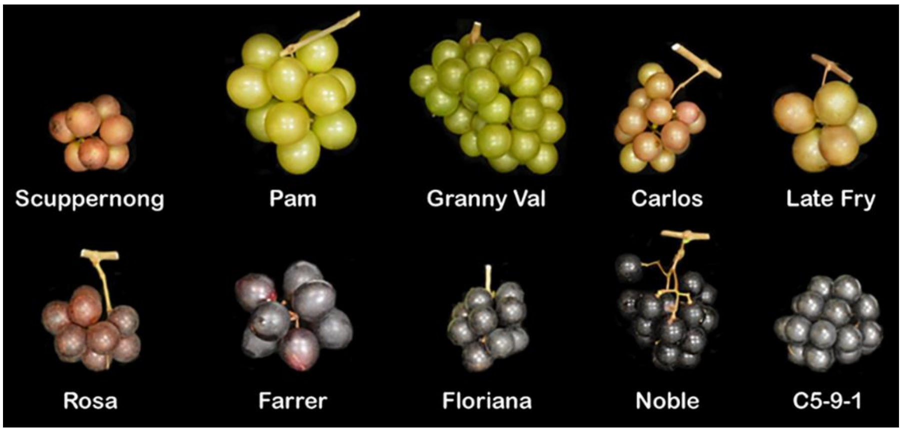

Supplementary Fig. 1. A representative image for muscadine genotypes used to determine berry color characteristics.

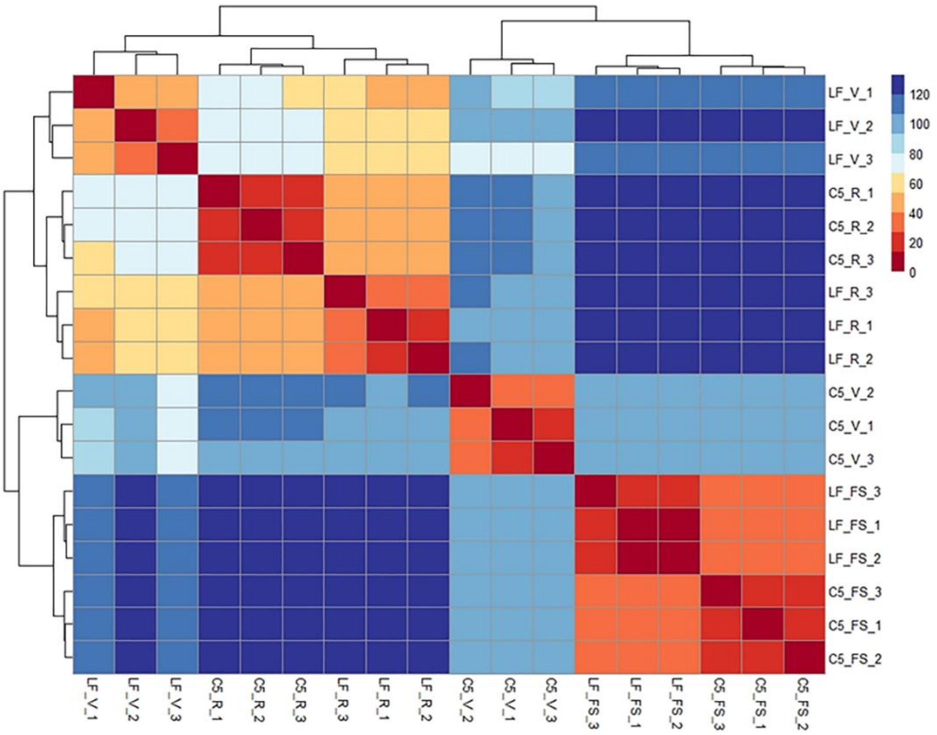

Supplementary Fig. 2. Heatmap of sample-to-sample distances of RNA-seq data from berries throughout developmental stages of the two muscadine genotypes (C5 and LF) using the variance stabilizing transformation (VST) and Euclidean distances. FS, fruit-set; V, véraison; R, ripening.

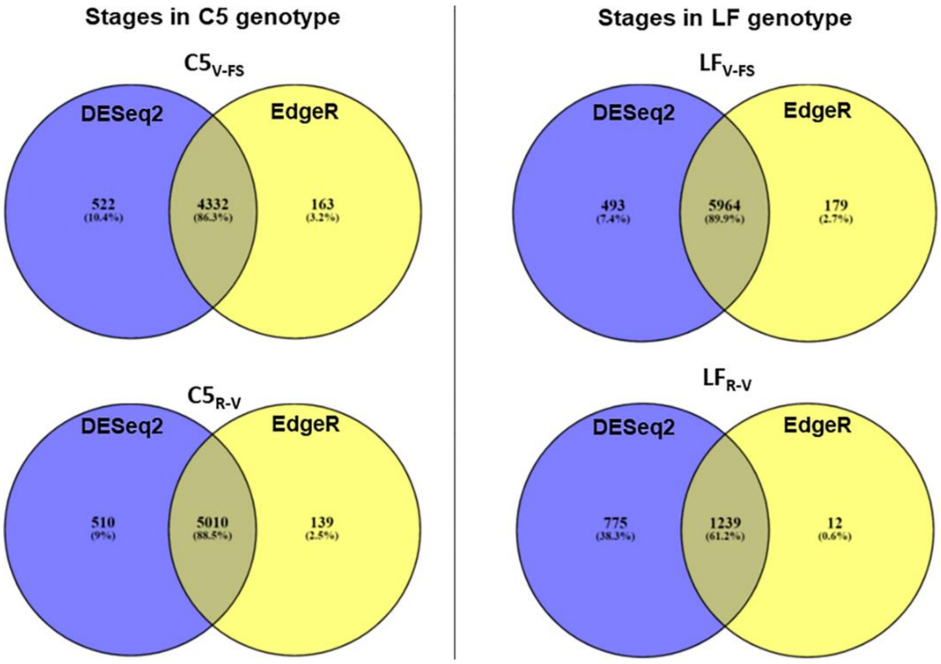

Supplementary Fig. 3. Venn diagrams of DEGs in muscadine C5 and LF genotypes. Genes that are differentially expressed when each developmental stage is compared to its earlier stage in C5 or LF genotypes using DESeq2 or EdgeR pipelines resulted in 8034 or 7412 non-redundant DEGs in C5 or LF, respectively. The fold2change of those genes is>1.5 or < –1.5. FS, fruit-set; V, véraison; R, ripening.

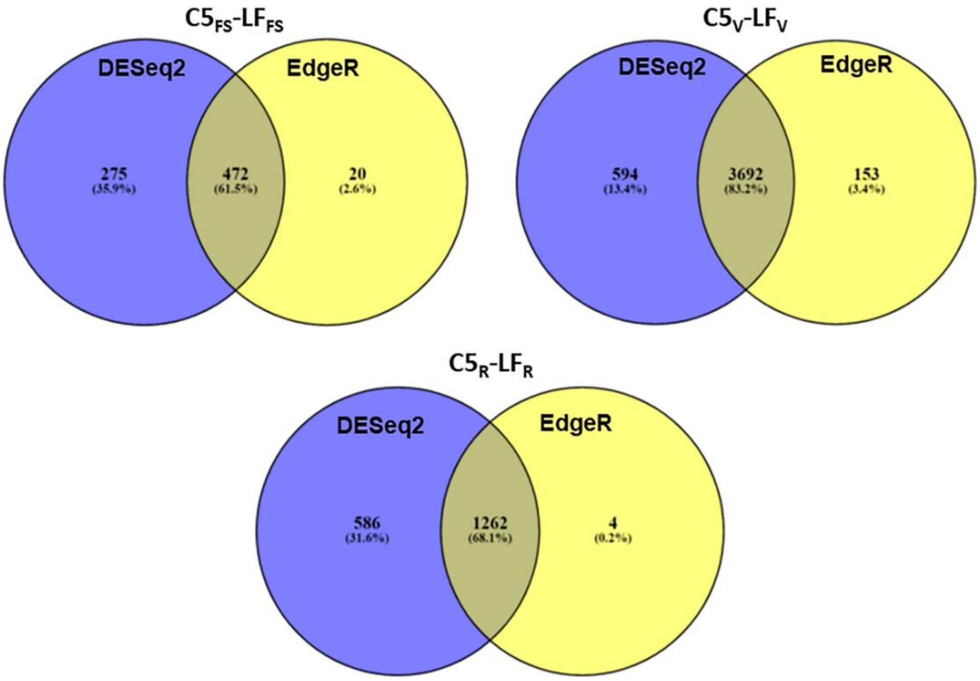

Supplementary Fig. 4. Venn diagrams of DEGs when muscadine C5 stages were compared to its corresponding LF stages ($C5_{stage}$-LF_{stage}), using DESeq2 or EdgeR pipelines resulted in non-redundant 5515 genes. The fold2change of those genes is>1.5 or <–1.5. FS, fruit set; V, véraison; R, ripening.

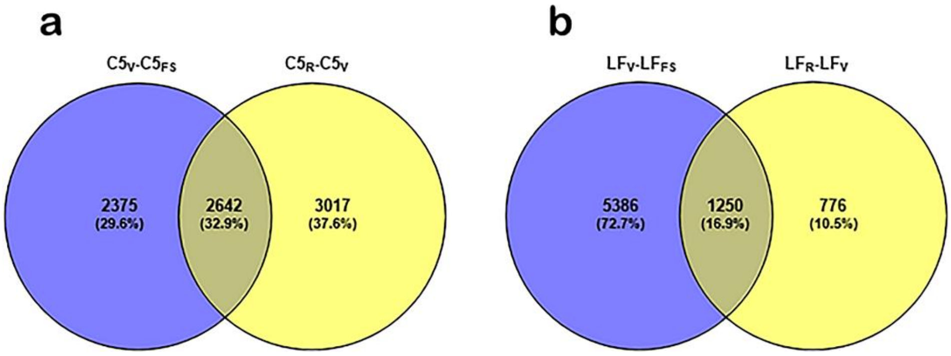

Supplementary Fig. 5. Venn diagrams of DEGs when berry developmental stage in muscadine genotypes, C5 or LF. By using DESeq2 (a) and EdgeR (b) pipelines, each stage was compared to its earlier one within the same genotype. As a result, 8034 and 7412 non-redundant DEGs were identified in C5 and LF, respectively, with fold2change >1.5 or < –1.5.

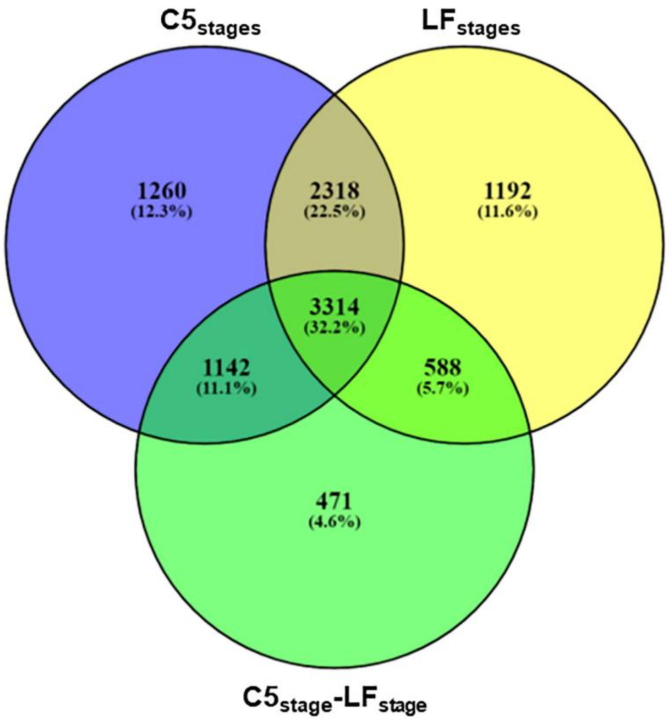

Supplementary Fig. 6. Venn diagrams of DEGs when stages in C5 or LF were compared against its earlier one within the same genotype or stage in C5 was compared against its corresponding in LF using DESeq2 and EdgeR pipelines. The fold2change of those genes is>1.5 or <–1.5. The resultant number of DEGs were 8034, 7412, or 5515 in C5, LF, or $C5_{stage}$-LF_{stage}, respectively.

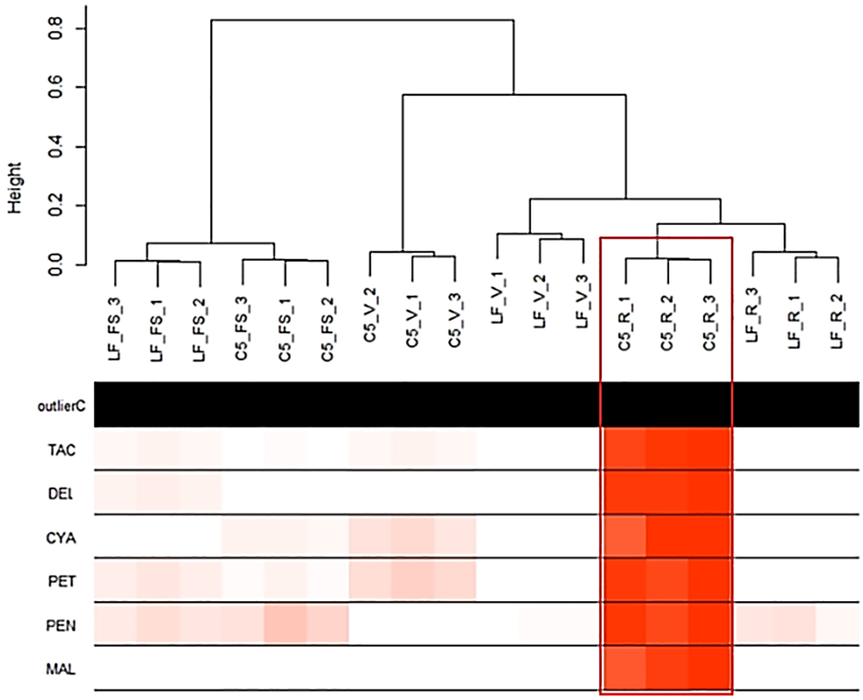

Supplementary Fig. 7. Clustering/sample dendrogram and trait heatmap of samples based on their Euclidean distance. Clustering dendrogram of 18 RNA-seq samples from muscadine berries during development based on their Euclidean distance. Trait heatmap shows the association of samples with the anthocyanin accumulation traits, including total anthocyanin content (TAC), delphinidin (DEL), cyanidin (CYA), petunidin (PET), peonidin (PEN), and malvidin (MAL) from C5 and LF genotypes at different berry developmental stages. White and red color represents low and high values, respectively.

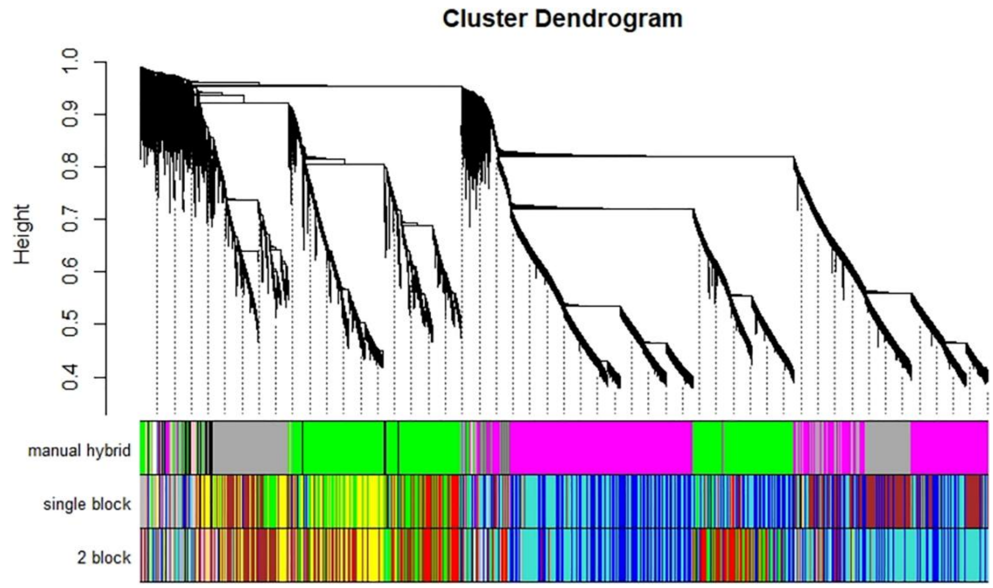

Supplementary Fig. 8. Clustering/sample dendrogram and trait heatmap of samples based on their Euclidean distance. Hierarchical cluster dendrogram exhibiting co-expressed modules identified by weighted gene co-expression network analysis for the muscadine C5 RNA-seq data. Each leaf on the tree represents one gene. The major tree branches constitute 14 merged modules with a threshold of 0.25 and different color labels. Clustering dendrogram of the 9 RNA-seq libraries from muscadine C5 berry during development based on their Euclidean distance. Trait heatmap shows the association of samples with anthocyanin-related traits, including total anthocyanin content (TAC), delphinidin (DEL), cyanidin (CYA), petunidin (PET), peonidin (PEN), and malvidin (MAL) from C5 genotype at different berry developmental stages. White and red color represents low and high values, respectively.

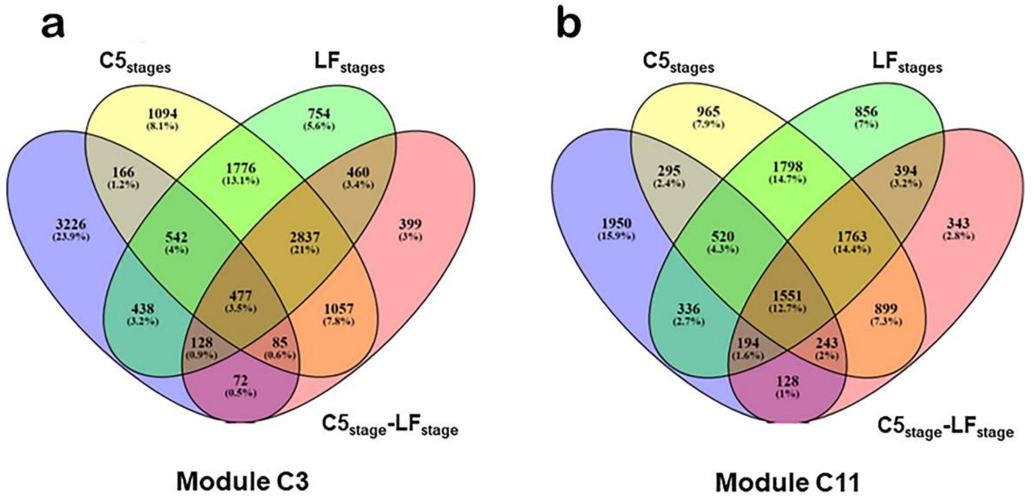

Supplementary Fig. 9. Venn diagrams of the genes distribution from C3 (a) and C11 (b) modules within the DEGs from $C5_{stages}$, LF_{stages}, and $C5_{stage}$-LF_{stage} comparisons.

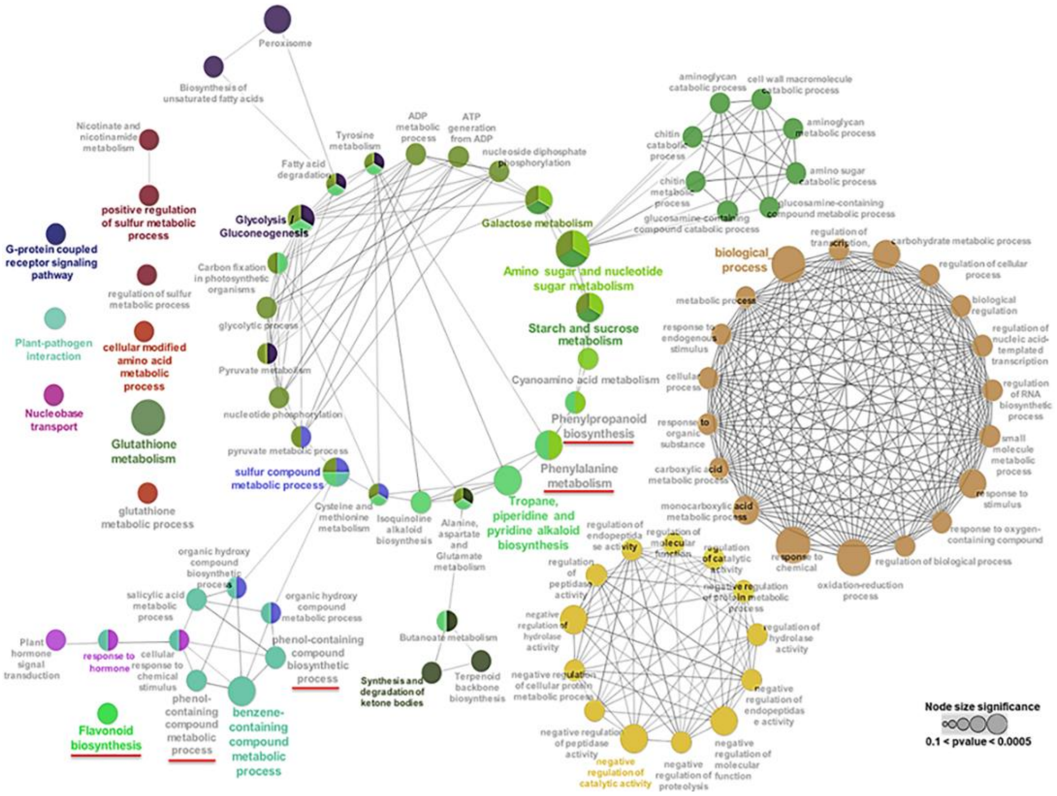

Supplementary Fig. 10. A network view for the predefined Biological processes GO terms and KEGG pathways in the 1908 DEGs of the positively correlated C3 module (p adjusted <0.05), extracted by g:Profiler website with Benjamini-Hochberg FDR multiple testing correction method. The default ClueGO settings were applied, and the terms are functionally grouped based on shared genes (kappa score). The most significant term defines the name of the group. The size of the nodes indicates the degree of significance with a p-value ranging from 0.05-0.005, 0.005-0.0005, and <0.0005. The interested BPs GO terms and KEGG pathways in C3 are underlined.

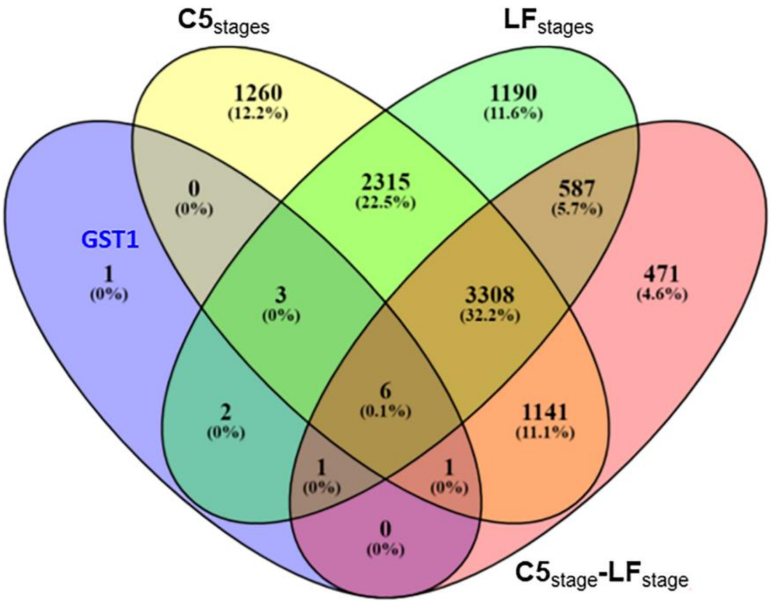

Supplementary Fig. 11. Venn diagrams of the distribution of the 14 candidate genes within the DEGs from $C5_{stages}$, LF_{stages}, and $C5_{stage}$-LF_{stage} comparisons. Out of the selected genes, only GST1 was not differentially expressed among stages; however, it was significantly expressed.

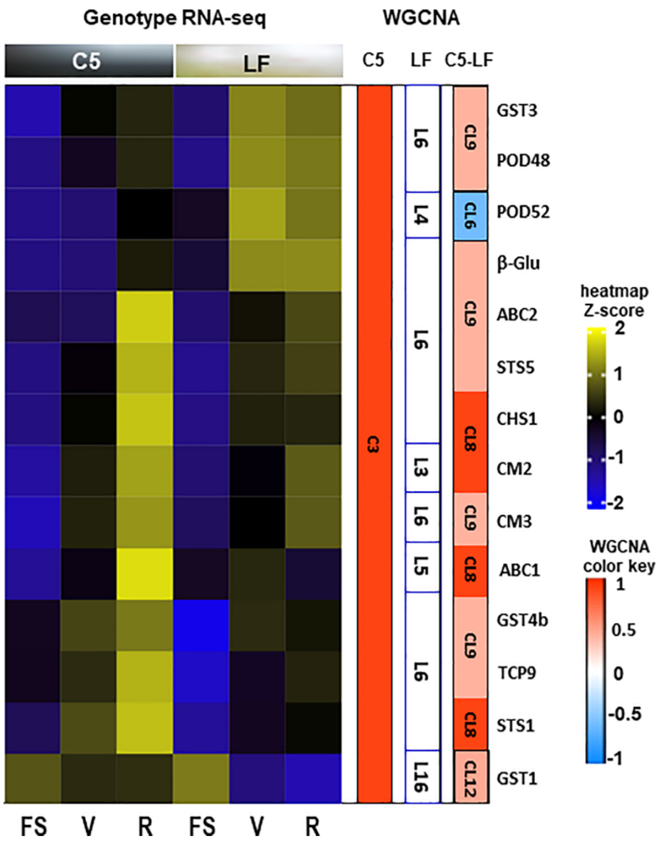

Supplementary Fig. 12. Heatmap of the expression values of the 14 candidate genes and their correlation to the trait data. Heatmap of the transcript per million (TPM) of the 14 candidate genes from C5 and LF genotypes at different developmental stages. In addition, their distribution in the module-trait correlation matrices that were constructed between RNA-seq data and evaluated traits, including TAC, DEL, CYA, PET, PEN, and MAL from C5, LF, or C5-LF data at different developmental stages. However, the correlation pattern of these 14 genes was not consistent in LF, as modules showed a positive correlation between some traits and a negative to others. Therefore, we did not assign a color to these modules (see Supplementary Fig. 13). Red and green are the Z-score value of the TPM values. Red and blue are the color key that represents r^2 values from –1 to 1.

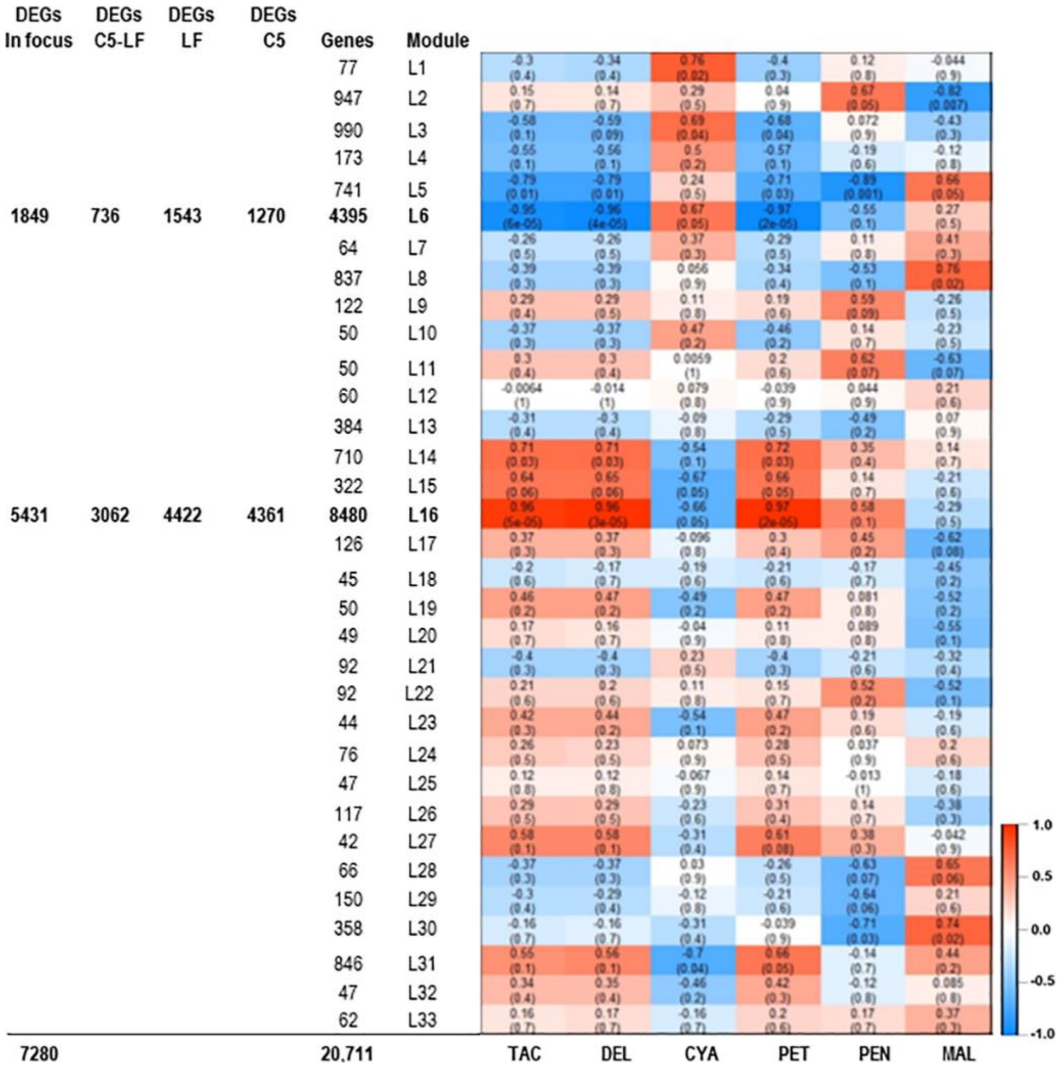

Supplementary Fig. 13. Module-trait associations between RNA-seq data and anthocyanin-related traits, including TAC, DEL, CYA, PET, PEN, and MAL from LF genotype at different berries developmental stages. The correlation coefficient between a given module and traits is indicated by the color of the cell at the row-column intersection. Each row corresponds to a module (L1-L33). The left panel shows the assigned number of genes to each module, whether the number of total input genes or DEGs from $C5_{stages}$, LF_{stages}, and $C5_{stage}$-LF_{stage} comparisons. The modules of interest were shown in bold and were selected for further analysis (indicated as 'In focus'). Blue and red are the color key that represents r^2 values from –1 to 1.

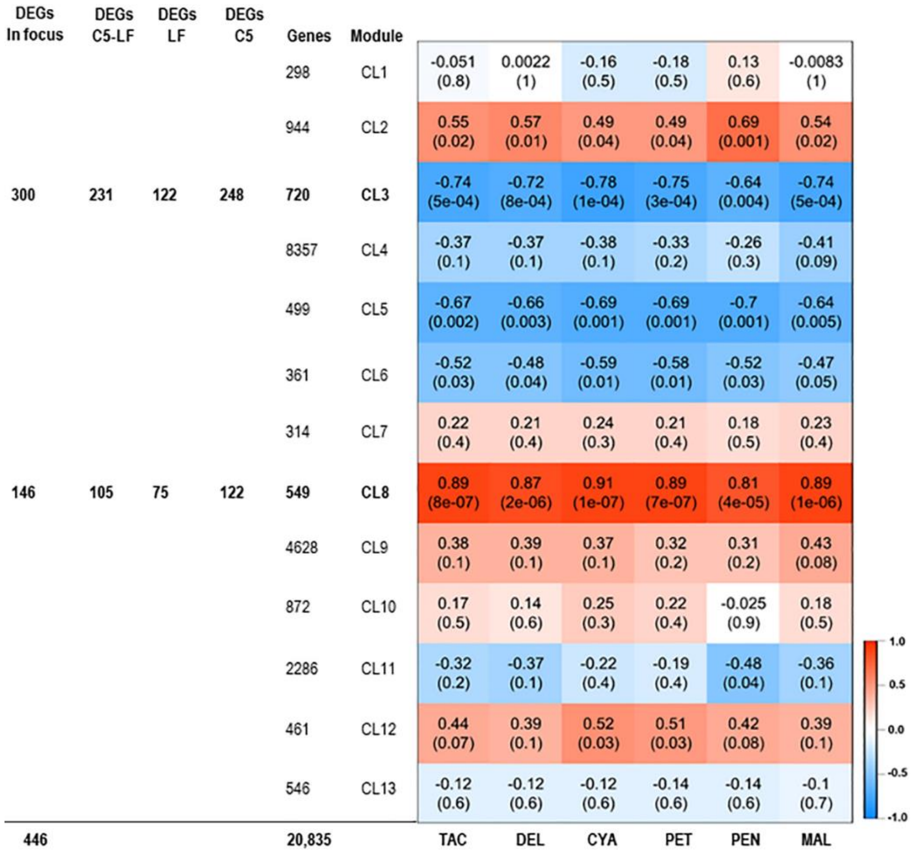

Supplementary Fig. 14. Module-trait associations between RNA-seq data and anthocyanin-related traits, including TAC, DEL, CYA, PET, PEN, and MAL from C5 and LF genotypes at different berries' developmental stages. The correlation coefficient between a given module and traits is indicated by the color of the cell at the row-column intersection. Each row corresponds to a module (CL1-CL13). The left panel shows the assigned number of genes to each module, whether the number of total input genes or DEGs from $C5_{stages}$, LF_{stages}, and $C5_{stage}$-LF_{stage} comparisons. The modules of interest were shown in bold and were selected for further analysis (indicated as 'In focus'). Blue and red are the color key that represents r^2 values from –1 to 1.

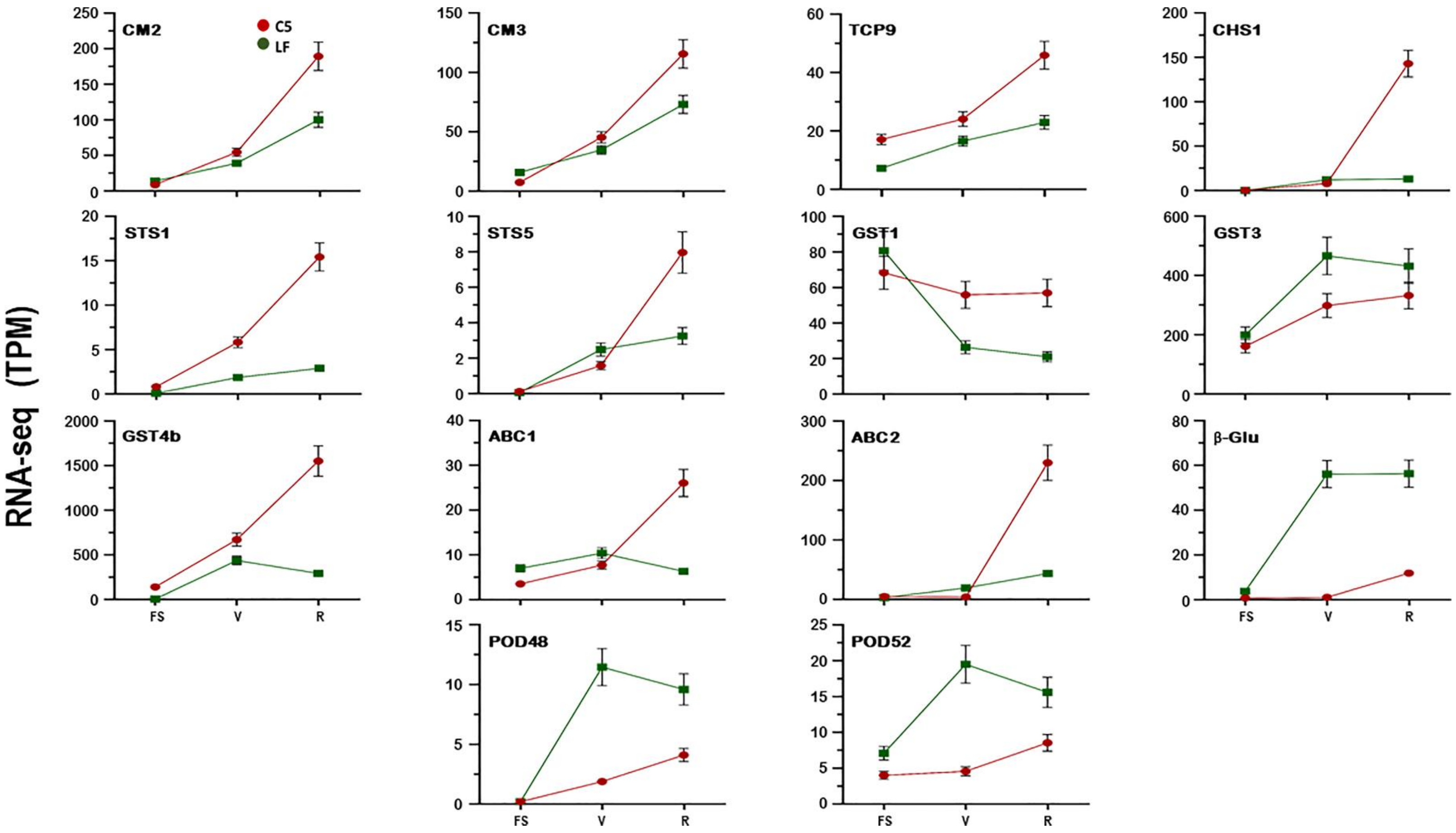

Supplementary Fig. 15. Comparison of expression profiles of fourteen DEGs from module C3 as measured by RNA-seq in C5 and LF genotypes at different berry developmental stages. The genes encode chorismate mutase (*CM2* and *CM3*), stilbene synthase (*STS1* and *STS5*), teosinte branched1/cincinnata/proliferating cell factor (*TCP9*), chalcone synthase (*CHS1*), glutathione S-transferase (*GST1*, *GST3*, and *GST4b*), ATP-binding cassette transporters (*ABC1* and *ABC2*), β-glucosidase (*β-Glu*), and peroxidase (*POD48* and *POD52*). The y-axis represent the mean expression level (±SD) determined by RNA-seq in TPM values calculated from three biological replicates (n=3). The x-axis in each chart represents the developmental stages.

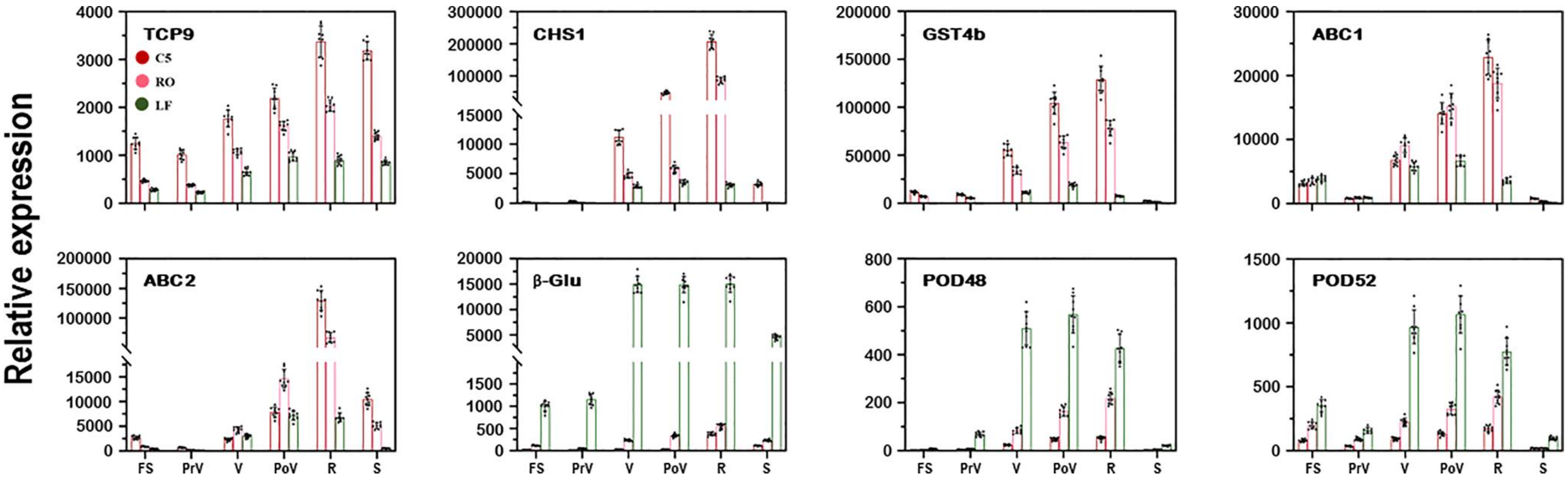

Supplementary Fig. 16. Steady-state transcript levels of anthocyanin-related genes identified by transcriptome analysis, including *TCP9*, *CHS1*, *GST4b*, *ABC1*, *ABC2*, *β-Glc*, *POD48*, and *POD52* mRNAs assessed by qPCR during berry development of muscadine genotypes C5-9-1 (C5, black berries), Rosa (Ro, red berries), and Late Fry (LF, bronze berries). The y-axis represents the mean expression level (±SD) from three biological and three technical replicates (n=9). The x-axis in each chart represents the developmental stages. Standard curves were used to calculate the number of target gene molecules per sample. These were then normalized relative to the expression of *Actin* and *EF1*.

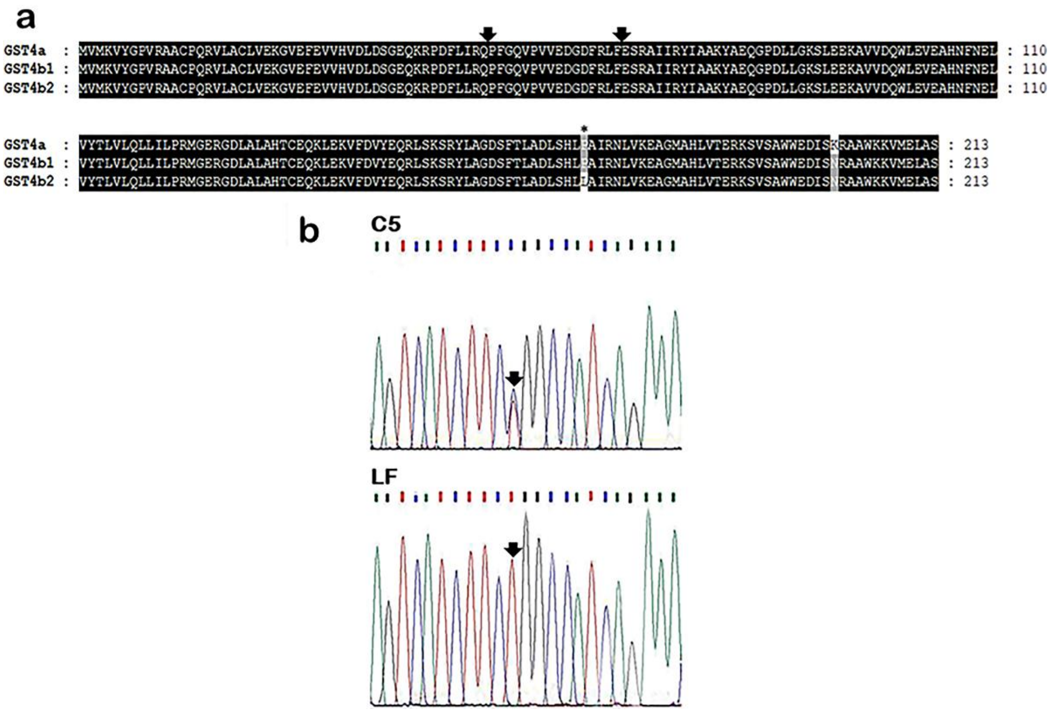

Supplementary Fig. 17. (**a**) Amino acid sequence alignment of muscadine GST4 proteins using ClustalX program. Conserved residues are shaded in black. Dark grey shading indicates similar residues in two out of three of the sequences. The GST4a associated with colored genotypes is absent in colorless muscadines. GST4b1 is associated with colored muscadines; however, the mutated GST4b2 is linked to colorless muscadines. The asterisk sign indicates the mutation position, where the amino acid residue P_{171} is changed to L. The black arrows indicate the positions of introns occurring among the various GST4 proteins. (**b**) Chromatograms of the nonsynonymous SNP region of the GST4b gene in the black (C5) and bronze (LF) genotypes. The C5 genotype holds the two different GST4b allele types (C : T); however, LF exhibited a homozygous T-allele. The black arrows indicate the position of the SNP.

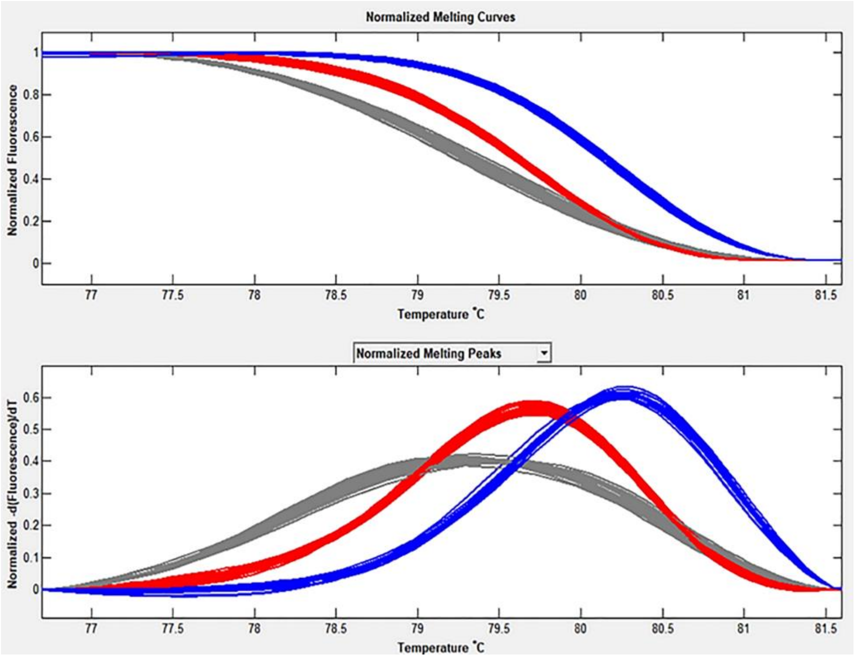

Supplementary Fig. 18. Discrimination of 328 muscadine genotypes for berry color trait using PCR strategy with GST4b gene and high-resolution melting (HRM) analysis. Representative profiles of the melting curves (difference plot curves) and normalized melting peaks of GST4b amplicons. The image showed three different allelic combinations of C : C (blue – homozygous), T : T (red – homozygous), and C : T (gray – heterozygous).

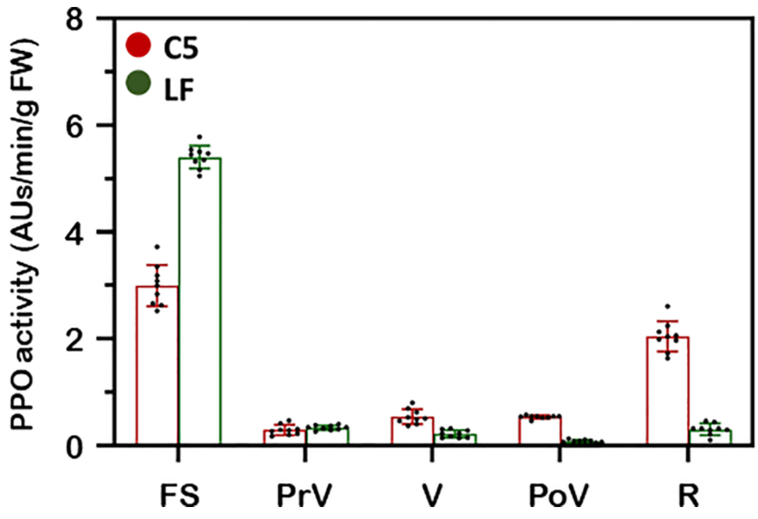

Supplementary Fig. 19. Enzymatic activity of PPO (AUs/ min/g FW) in muscadine berry skin during C5 and LF berry development. The y-axis represents the mean activity level (±SD) from three biological and three technical replicates (n=9). The x-axis represents the developmental stages.

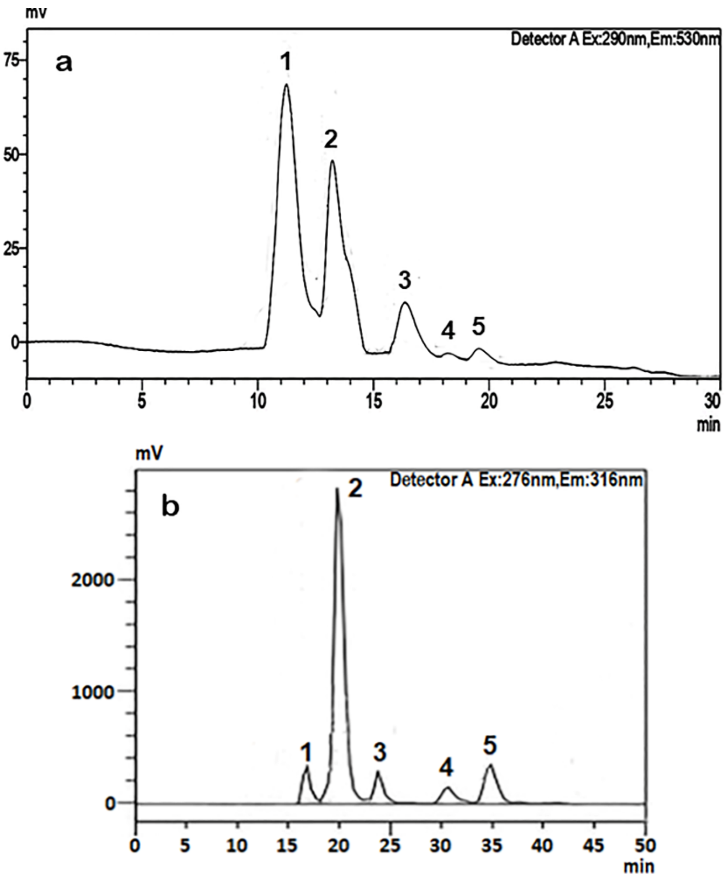

Supplementary Fig. 20. (**a**) HPLC chromatogram traces of C5 mature skin sample. Peaks of individual anthocyanins were annotated as follows peak-1 is delphinidin; peak-2 is cyanidin; peak-3 is petunidin; peak-4 is peonidin, and peak-5 is malvidin. (**b**) HPLC chromatogram traces of LF mature skin sample. Peaks of individual PAs were annotated as follows peak-1 is epigallocatechin cinnamate, peak-2 is (+)-catechin, peak-3 is procyanidin B1, peak-4 is (-)-epicatechin gallate, and peak-5 is procyanidin C1. mV: millivolt.
