# Supplementary material for: A recessive mutation in muscadine grapes causes berry color-loss without influencing anthocyanin pathway
Source: Commun Biol. 2022 Sep 24;5:1012. doi: 10.1038/s42003-022-04001-8 (PMC9509324; doi:10.1038/s42003-022-04001-8)
Supplement: Supplementary file 2 — Description of Additional Supplementary Files [file 42003_2022_4001_MOESM2_ESM.pdf]

Description of Additional Supplementary Files

File name: Supplementary Data 1
Description: Berry color phenotype and HRM predicted GST4b allelotype of 272 and 56 muscadine breeding lines and cultivars, respectively.

File name: Supplementary Data 2
Description: Summary of read mapping rate of RNA-Seq libraries of C5 and LF muscadine berries at different developmental stages; fruit-set (FS), véraison (V), and ripening (R).

File name: Supplementary Data 3
Description: Differentially expressed genes over time course of C5 muscadine berry throughout development.

File name: Supplementary Data 4
Description: Differentially expressed genes over time course of LF muscadine berry throughout development.

File name: Supplementary Data 5
Description: Differentially expressed genes when muscadine C5 berry stages were compared to its corresponding LF berry stages.

File name: Supplementary Data 6
Description: Module-trait association analysis between the RNA-seq data and the C5 anthocyanin-related data in the modules of interest; C3 and C11.

File name: Supplementary Data 7
Description: The GO and KEGG enrichment in the modules of interest; C3 and C11, based on the V. vinifera Ensembl Gene ID.

File name: Supplementary Data 8
Description: (a) The oligonucleotide primers of assessed genes by qPCR. The correlation between RNA-seq of C5 genotype data and the evaluated traits. (b) The oligonucleotide primers used for GST4b genotyping by HRM analysis.

File name: Supplementary Data 9
Description: The GO and KEGG enrichment in the 14 DEGs of interest based on the V. vinifera Ensembl Gene ID.

File name: Supplementary Data 10
Description: List of genes identified between flanking markers of chr4_11238026.
