# Supplementary material for: A recessive mutation in muscadine grapes causes berry color-loss without influencing anthocyanin pathway
Source: Commun Biol. 2022 Sep 24;5:1012. doi: 10.1038/s42003-022-04001-8 (PMC9509324; doi:10.1038/s42003-022-04001-8)
Supplement: Supplementary file 13 — Reporting Summary [file 42003_2022_4001_MOESM13_ESM.pdf]

nature portfolio

Corresponding author(s): *Double-blind peer review submissions: write DBPR and your manuscript number here instead of author names.*

Last updated by author(s): *YYYY-MM-DD*

Reporting Summary

Nature Portfolio wishes to improve the reproducibility of the work that we publish. This form provides structure for consistency and transparency in reporting. For further information on Nature Portfolio policies, see our Editorial Policies and the Editorial Policy Checklist.

Statistics

For all statistical analyses, confirm that the following items are present in the figure legend, table legend, main text, or Methods section.

n/a	Confirmed	
☐	☒	The exact sample size (*n*) for each experimental group/condition, given as a discrete number and unit of measurement
☐	☒	A statement on whether measurements were taken from distinct samples or whether the same sample was measured repeatedly
☐	☒	The statistical test(s) used AND whether they are one- or two-sided<br>*Only common tests should be described solely by name; describe more complex techniques in the Methods section.*
☐	☒	A description of all covariates tested
☐	☒	A description of any assumptions or corrections, such as tests of normality and adjustment for multiple comparisons
☐	☒	A full description of the statistical parameters including central tendency (e.g. means) or other basic estimates (e.g. regression coefficient) AND variation (e.g. standard deviation) or associated estimates of uncertainty (e.g. confidence intervals)
☐	☒	For null hypothesis testing, the test statistic (e.g. *F*, *t*, *r*) with confidence intervals, effect sizes, degrees of freedom and *P* value noted<br>*Give P values as exact values whenever suitable.*
☒	☐	For Bayesian analysis, information on the choice of priors and Markov chain Monte Carlo settings
☒	☐	For hierarchical and complex designs, identification of the appropriate level for tests and full reporting of outcomes
☐	☒	Estimates of effect sizes (e.g. Cohen's *d*, Pearson's *r*), indicating how they were calculated

Our web collection on statistics for biologists contains articles on many of the points above.

Software and code

Policy information about availability of computer code

Data collection	All data have been collected, saved, and presented as supplemental tables in excel files.
Data analysis	Sigma Plot, Students' T-test, FASTQ, Trimmomatic (v0.39), Edge R pipelines, DESeq2 pipelines, web-based Venny tool, R package WGCNA (v1.69), g:Profiler website, Clue GO, TASSEL v5.2.79 software, MODELLER package, LeDock software, regression, correlation coefficient.

For manuscripts utilizing custom algorithms or software that are central to the research but not yet described in published literature, software must be made available to editors and reviewers. We strongly encourage code deposition in a community repository (e.g. GitHub). See the Nature Portfolio guidelines for submitting code & software for further information.

Data

Policy information about availability of data

All manuscripts must include a data availability statement. This statement should provide the following information, where applicable:

- Accession codes, unique identifiers, or web links for publicly available datasets
- A description of any restrictions on data availability
- For clinical datasets or third party data, please ensure that the statement adheres to our policy

All RNA-seq data generated during the current study are available in the NCBI GenBank: PRJNA775666 and PRJNA810835.

Human research participants

Policy information about studies involving human research participants and Sex and Gender in Research.

Reporting on sex and gender	Not applicable
Population characteristics	Not applicable
Recruitment	Not applicable
Ethics oversight	Not applicable

Note that full information on the approval of the study protocol must also be provided in the manuscript.

Field-specific reporting

Please select the one below that is the best fit for your research. If you are not sure, read the appropriate sections before making your selection.

☒ Life sciences ☐ Behavioural & social sciences ☐ Ecological, evolutionary & environmental sciences

For a reference copy of the document with all sections, see nature.com/documents/nr-reporting-summary-flat.pdf

Life sciences study design

All studies must disclose on these points even when the disclosure is negative.

Sample size	For any biological study analysis (i.e., metabolites quantification, enzymatic activity, and qPCR), the experiment was conducted using three biological and technical replicates and the data were presented as an average of nine replicates (±SD). The RNA-seq data were generated from three biological replicates. For GWAS study, the population was represented by 348 individual muscadine genotypes. For the HRM analysis, the study was conducted using 328 individual muscadine genotypes.
Data exclusions	The expression of GST12 gene found within the QTL region highlighted by GWAS analysis was not detected in different muscadine tissues (i.e., root, leaves, flower, and tendrils) or during muscadine berry development as determined by RNA-seq and qPCR assay approaches. Accordingly, we mentioned in the manuscript the next statement "Analysis of RNA-seq data among various tissues and berry developmental stages indicated that GST12a/b genes are not expressed (data not shown). Consequently, no further analysis was performed for these two genes".
Replication	For any biological study analysis (i.e., metabolites quantification, enzymatic activity, and qPCR), the experiment was conducted using three biological and technical replicates and the data were presented as an average of nine replicates (±SD). The RNA-seq data were generated from three biological replicates. The RNA-seq data were analyzed using two different pipelines, Edge R and DESeq2, to validate the analysis. Further, we strongly believe that the number of individual muscadine genotypes used for GWAS and HRM analysis are largely sufficient to validate related studies.
Randomization	The data related to population characterization for anthocyanin levels were assessed for three consecutive years. All muscadine vines included in this study were represented by three copy vines that are located in different places within the vineyard through which each vine represent a biological replicate. Samples exhibiting similar developmental stage (age) from different locations within the same vine were collected (technical replicate). This strategy allowed us to ensure the reproductivity of the results, irrespective to the potential involvement of environmental and management factors.
Blinding	Yes the researchers collected the samples only with the essential requirement, i.e. correct genotype and correct developmental stage.

Reporting for specific materials, systems and methods

We require information from authors about some types of materials, experimental systems and methods used in many studies. Here, indicate whether each material, system or method listed is relevant to your study. If you are not sure if a list item applies to your research, read the appropriate section before selecting a response.

Materials & experimental systems

n/a	Involved in the study
☒	☐ Antibodies
☒	☐ Eukaryotic cell lines
☒	☐ Palaeontology and archaeology
☒	☐ Animals and other organisms
☒	☐ Clinical data
☒	☐ Dual use research of concern

Methods

n/a	Involved in the study
☒	☐ ChIP-seq
☒	☐ Flow cytometry
☒	☐ MRI-based neuroimaging
